# Limb‐sparing surgery plus radiotherapy results in superior survival: an analysis of patients with high‐grade, extremity soft‐tissue sarcoma from the NCDB and SEER

**DOI:** 10.1002/cam4.1625

**Published:** 2018-07-20

**Authors:** Stephen J. Ramey, Raphael Yechieli, Wei Zhao, Joyson Kodiyan, David Asher, Felix M. Chinea, Vivek Patel, Isildinha M. Reis, Lily Wang, Breelyn A. Wilky, Ty Subhawong, Jonathan C. Trent

**Affiliations:** ^1^ Department of Radiation Oncology University of Miami Sylvester Comprehensive Cancer Center Miami FL USA; ^2^ Biostatistics and Bioinformatics Shared Resources (BBSR) University of Miami Sylvester Comprehensive Cancer Center Miami FL USA; ^3^ Department of Internal Medicine Jackson Health System Miami FL USA; ^4^ Department of Radiation Oncology Holy Cross Hospital Fort Lauderdale FL USA; ^5^ Division of Biostatistics Department of Public Health Sciences University of Miami School of Medicine Miami FL USA; ^6^ Division of Medical Oncology Department of Medicine University of Miami Sylvester Comprehensive Cancer Center Miami FL USA; ^7^ Department of Radiology University of Miami School of Medicine Miami FL USA

**Keywords:** amputation, limb‐sparing surgery, National Cancer Database, NCDB, radiotherapy, sarcoma, SEER program, survival

## Abstract

Small randomized trials have not shown an overall survival (OS) difference among local treatment modalities for patients with extremity soft‐tissue sarcomas (E‐STS) but were underpowered for OS. We examine the impact of local treatment modalities on OS and sarcoma mortality (SM) using two national registries. The National Cancer Database (NCDB) and the Surveillance, Epidemiology, and End Results (SEER) Program were analyzed separately to identify patients with stage II‐III, high‐grade E‐STS diagnosed between 2004 and 2013 and treated with (1) amputation alone, (2) limb‐sparing surgery (LSS) alone, (3) preoperative radiation therapy (RT) and LSS, or (4) LSS and postoperative RT. Multivariable analyses (MVAs) and 1:1 matched pair analyses (MPAs) examined treatment impacts on OS (both databases) and SM (SEER only). From the NCDB and SEER, 7828 and 2937 patients were included. On MVAs, amputation was associated with inferior OS and SM. Relative to LSS alone, both preoperative RT and LSS (HR, 0.70; 95% CI: 0.62‐0.78) and LSS and postoperative RT (HR, 0.69; 95% CI: 0.63‐0.75) improved OS in NCDB analyses with confirmation by SEER. Estimated median survivals from MPA utilizing NCDB data were 7.2 years with LSS alone (95% CI: 6.5‐8.9 years) vs 9.8 years (95% CI: 9.0‐11.2 years) with LSS and postoperative RT. A MPA comparing preoperative RT and LSS to LSS alone found median survivals of 8.9 years (95% CI: 7.9‐not estimable) and 6.6 years (95% CI: 5.4‐7.8 years). Optimal high‐grade E‐STS management includes LSS with preoperative or postoperative RT as evidenced by superior OS and SM.

## 1 INTRODUCTION

Extremity soft‐tissue sarcoma (E‐STS) is a rare malignancy accounting for 0.7% of cancer diagnoses in 2017.^1^ Limb‐sparing surgery (LSS) combined with radiation therapy (RT) has become the preferred treatment regimen^2^ for high‐grade E‐STS since a randomized trial showed similar disease‐free survival and overall survival (OS) to amputation.^3^ Subsequent trials have shown greater local control (LC) for high‐grade E‐STS patients receiving LSS and RT. Although no difference in OS was seen, these studies were underpowered for all but very large differences in OS.^4^, ^5^ A trial comparing preoperative RT to postoperative RT found similar LC and OS.^6^, ^7^

Previous large dataset analyses of the impact of local treatment on survival outcomes for E‐STS had inconsistent results and some important limitations. Two National Cancer Database (NCDB)^8^, ^9^ and two Surveillance, Epidemiology, and End Results (SEER) studies^10^, ^11^ indicated a survival benefit using RT in the treatment of high‐grade E‐STS. In contrast, three other SEER studies showed either no survival benefit for RT or a benefit only in patients with tumors >5 cm.^12^, ^13^, ^14^, ^15^ Importantly, most of these studies did not contain data from the last decade,^8^, ^10^, ^12^, ^13^, ^14^, ^15^ and all except two^8^, ^14^ excluded patients treated with amputation or did not specify type of resection.^9^ Several SEER^10^, ^11^ or smaller retrospective studies^16^, ^17^ found no difference in survival or LC between preoperative and postoperative RT. In contrast, a study from the National Oncology Database found significantly greater OS and cause‐specific survival with preoperative RT.^18^

Herein, we provide a more comprehensive and robust overview of the impact of local treatment on survival outcomes. In contrast to previously published large cancer registry studies, we have selected a more contemporary cohort of E‐STS patients without exclusions based on definitive surgical technique. Additionally, to our knowledge, this is the first sarcoma study to utilize both the SEER and NCDB registries and the first to investigate the impact of the four common local treatment options (amputation, LSS alone, LSS and postoperative RT, and preoperative RT and LSS) on the survival of patients with high‐grade E‐STS in a single analysis. By analyzing recent data from both databases with a similar selection criteria and statistical methodology, this study aims to clarify the impact of local treatment modalities on high‐grade E‐STS survival.

## 2 METHODS

The Human Subjects Research Office at the University of Miami certified that this study was exempt from IRB review. SEER data, which includes about 30% of the US patient population, were obtained from the November 2015 submission; access to the 2014 NCDB participant user file was granted by the American College of Surgeons. More than 1500 Commission on Cancer (CoC)‐accredited cancer programs currently submit data to the NCDB on approximately 70% of new cancer cases in the United States,^19^ including approximately 78% of soft‐tissue malignancies.^20^ The NCDB is a joint project of the CoC of the American College of Surgeons and the American Cancer Society. The CoC's NCDB and the hospitals participating in the CoC NCDB are the source of the de‐identified data used herein; they have not verified and are not responsible for the statistical validity of the data analysis or the conclusions derived by the authors. Data reported to the NCDB undergo a battery of tests and audits to ensure the accuracy and completeness of the data.^21^, ^22^

Of note, no attempt was made to combine data from these two datasets. Combining data from NCDB and SEER would be inappropriate since there is likely significant overlap between patients reported to these two databases, and there is no way to delineate when such overlap exists. Therefore, the two datasets were analyzed in parallel.

### 2.1 Patient population

An initial cohort of 82 987 patients diagnosed between 2004 and 2014 was received from the NCDB. Exclusion criteria applied are documented in Figure S1. Briefly, only patients with high‐grade E‐STS, American Joint Committee on Cancer (AJCC) stage II‐III without nodal involvement, no history of prior malignancy, and definitive intent treatment were included. Eligible patients were treated with (1) amputation alone, (2) LSS alone, (3) preoperative RT and LSS, or (4) LSS and postoperative RT. Patients with non‐standard RT modalities for high‐grade E‐STS (eg, orthovoltage, electrons only, etc.) were excluded. Finally, patients without follow‐up regarding vital status were also excluded, resulting in exclusion of all patients diagnosed in 2014. Similar exclusion criteria were applied for the SEER data (Figure S2).

### 2.2 Study variables

All variables included in Table S1 were utilized in all NCDB analyses. Race and ethnicity were combined into one composite variable. Data on median household income and high school graduation rates within the patient's zip code at diagnosis were derived from the 2012 American Community Survey. Urban/rural status was defined using the 2013 files published by the US Department of Agriculture Economic Research Service. A “transition in care” occurred when the NCDB reported that a patient received care at more than one center during the diagnosis and treatment processes. Charlson/Deyo Scores were used to assess comorbidity. The NCDB collapses all scores >1 into a single category of “2” given the limited number of patients with values above 1. Facility volume was defined using facility identifier codes. Cutoffs of ≤7, 8‐81, and >81 included patients reported by the center were used to result in patient cohorts of approximately 25%, 50%, and 25% reported by low, intermediate, and high volume centers. Specifically, 25.2%, 50.2%, and 24.6% of patients were treated at low‐, mid‐, and high‐volume centers based on these cutoffs (Table S1). For NCDB analyses, AJCC stage was reported using the “analytic stage group” which utilizes the pathologic stage whenever reported and the clinical stage when pathologic stage is unavailable. Stage groups were based on the AJCC edition being used at the time of the patient's diagnosis (ie, 6th edition for patients diagnosed between 2004 and 2009 and 7th edition for patients diagnosed between 2010 and 2014).

All variables listed in Table S2 were utilized in SEER analyses. By design, these variables were similar to those utilized for the NCDB analyses. Median household income, educational attainment, and urban/rural status were derived as described above. Marital status was available in SEER but not in NCDB whereas comorbidity, distance to reporting center, facility type, reporting center sarcoma treatment volume, transitions in care, chemotherapy use, and surgical margin status were not available in SEER. The AJCC 6th edition was used for stage group in SEER as it was more widely reported than the 7th edition stage. To derive four regional groups for both the NCDB and SEER, geographic locations were grouped as follows from each database (postal codes shown in parentheses): Northeast (CT, MA, ME, NH, RI, VT, NJ, NY, PA), South (DC, DE, FL, GA, MD, NC, SC, VA, WV, AL, KY, MS, TN, AR, LA, OK, TX), Midwest (IL, IN, MI, OH, WI, IA, KS, MN, MO, ND, NE, SD), and West (AZ, CO, ID, MT, NM, NV, UT, WY, AK, CA, HI, OR, WA). Of note, the SEER database does not include data from all states.

### 2.3 Statistical analyses

Distributions of selected demographic and prognostic variables were compared by treatment using the chi‐square test. OS was defined as time from diagnosis to death from any cause with surviving patients censored at date of last follow‐up. OS curves were estimated using the Kaplan‐Meier method and compared using the log‐rank test. Multivariable analyses (MVAs) using Cox proportional hazards regression modeling were conducted to evaluate the effect of treatment on survival outcomes.^23^ Sarcoma mortality (SM) cumulative incidence curves were estimated and tested using the method by Gray.^24^ The effect of potential prognostic variables on SM was evaluated using the modified Cox proportional hazards model by Fine and Gray for competing risk data.^25^

For each dataset, treatment effects were first compared in MVA with all four possible treatments included. Subsequent MVAs were then performed to specifically compare survival with (1) preoperative RT and LSS vs LSS and postoperative RT, (2) LSS alone vs LSS with RT (either preoperative or postoperative), and (3) amputation vs LSS (with or without RT). Finally, one‐to‐one propensity score matching^26^ was performed to evaluate (1) preoperative RT followed by LSS vs LSS alone and (2) LSS followed by postoperative RT vs LSS alone to reduce the selection bias due to sampling. All tests were two‐sided. Statistical analyses were conducted using SAS software version 9.4 (SAS Institute Inc., Cary, NC).

## 3 RESULTS

### 3.1 Entire cohorts

From the NCDB data (Table S1), 7828 met inclusion criteria with 477 (6.1%) treated with amputation alone, 2203 (28.1%) with LSS alone, 1589 (20.3%) with preoperative RT and LSS, and 3559 (45.5%) with LSS and postoperative RT. Among 2937 patients included from SEER (Table S2), 168 (5.7%) were treated with amputation; 775 (26.4%) with LSS alone; 484 (16.5%) with pre‐RT and LSS; and 1510 (51.4%) with LSS and post‐RT.

**Figure 1:**
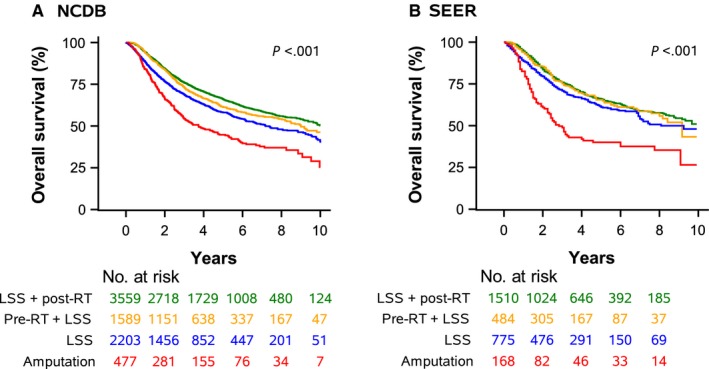
A. National Cancer Database (NCDB) Overall Survival by Treatment. B. Surveillance, Epidemiology, and End Results (SEER) Overall Survival by Treatment

On MVA (Table 1 for NCDB analysis and Table 2 for SEER analysis), amputation had significantly inferior OS compared to LSS alone (hazard ratio [HR], 1.31; 95% confidence interval [CI]: 1.13‐1.51 for NCDB and HR, 1.59; 95% CI: 1.24‐2.04 for SEER). SM was also increased with amputation in the SEER analysis (HR, 1.52; 95% CI: 1.12‐2.07). Preoperative RT was associated with superior OS relative to LSS alone in both datasets (HR, 0.70; 95% CI: 0.62‐0.78 for NCDB and HR, 0.60; 95% CI: 0.48‐0.75 for SEER). Preoperative RT followed by LSS was associated with lower SM than LSS alone using SEER data (HR, 0.75; 95% CI: 0.58‐0.97). Similarly, LSS combined with postoperative RT was also associated with superior OS relative to LSS alone in both NCDB (HR, 0.69; 95% CI: 0.63‐0.75) and SEER data (HR, 0.70; 95% CI: 0.60‐0.82). SM was also lower with postoperative RT compared to LSS alone (HR, 0.82; 95% CI: 0.67‐0.99). Kaplan‐Meier estimates of OS for all four treatment regimens are shown in Figure 1. When comparing preoperative RT and LSS to LSS and postoperative RT directly (analyses not shown), no significant difference in OS (HR, 1.01; 95% CI: 0.91‐1.13 for NCDB and HR, 0.86; 95% CI: 0.70‐1.06 for SEER) or SM (HR, 0.92; 95% CI: 0.73‐1.15) was found.

**Table 1:** Multivariate Cox regression on overall survival using National Cancer Database

Variable	Category	HR (95% CI)	*P*‐value
Treatment	LSS	Reference	
	Amputation	1.31 (1.13, 1.51)	<.001
	Pre‐RT + LSS	0.70 (0.62, 0.78)	<.001
	LSS + post‐RT	0.69 (0.63, 0.75)	<.001
Facility type	Community Program	Reference	
	Academic/Research Program	1.07 (0.97, 1.20)	.187
	Integrated Network Cancer Program	1.10 (0.95, 1.28)	.190
	Other/Unknown	0.78 (0.47, 1.28)	.319
Facility location	Northeast	Reference	
	South	0.91 (0.81, 1.02)	.106
	Midwest	1.00 (0.89, 1.13)	.990
	West	0.95 (0.83, 1.08)	.441
	Other/Unknown	NE	
Age at diagnosis	≤40	Reference	
	41‐50	0.87 (0.54, 1.41)	.577
	51‐60	1.01 (0.63, 1.61)	.978
	61‐70	1.05 (0.66, 1.70)	.827
	>70	1.86 (1.15, 2.99)	.011
Sex	Male	Reference	
	Female	0.86 (0.80, 0.92)	<.001
Race/ethnicity	White Non‐Hispanic	Reference	
	White Hispanic	0.92 (0.77, 1.10)	.345
	Black	1.07 (0.94, 1.22)	.320
	Other/Unknown	1.00 (0.88, 1.13)	.953
Insurance	Private Insurance	Reference	
	Not Insured	1.50 (1.23, 1.84)	<.001
	Medicaid	1.39 (1.17, 1.66)	<.001
	Medicare	1.32 (1.18, 1.49)	<.001
	Other/Unknown	1.14 (0.90, 1.44)	.276
Median income	≥$63 000	Reference	
	$48 000‐$62 999	0.96 (0.86, 1.07)	.460
	$38 000‐$47 999	0.92 (0.81, 1.06)	.246
	<$38 000	1.04 (0.89, 1.22)	.587
	Unknown	1.11 (0.41, 2.98)	.835
Educational: Non‐High school	<7%	Reference	
	7%‐12.9%	1.11 (0.99, 1.24)	.069
	13%‐20.9%	1.18 (1.03, 1.34)	.015
	≥21%	1.29 (1.10, 1.51)	.001
	Not available	4.52 (1.28, 15.9)	.019
Living location	Metro area	Reference	
	Smaller metro area	1.19 (1.08, 1.31)	<.001
	Urban area	1.05 (0.92, 1.21)	.441
	Rural area	1.27 (0.93, 1.73)	.140
	Unknown	1.03 (0.81, 1.31)	.810
Distance in miles to hospital	≤10	Reference	
	11‐20	1.11 (0.99, 1.23)	.069
	21‐50	1.14 (1.02, 1.27)	.025
	>50	1.02 (0.90, 1.16)	.706
	Unknown	0.64 (0.28, 1.47)	.295
Comorbidity	0	Reference	
	1	1.22 (1.11, 1.34)	<.001
	2	1.44 (1.21, 1.72)	<.001
Transition in care	No	Reference	
	Yes	0.84 (0.73, 0.96)	.012
	Unknown	0.83 (0.71, 0.97)	.021
Primary tumor site	Upper limb	Reference	
	Lower limb	1.01 (0.92, 1.11)	.827
Tumor size (cm)	≤5	Reference	
	5.01‐10	1.57 (1.38, 1.80)	<.001
	10.01‐15	2.15 (1.85, 2.49)	<.001
	>15	2.68 (2.31, 3.10)	<.001
Clinical tumor stage	I	Reference	
	II	1.29 (1.11, 1.50)	.001
	Unknown	1.27 (1.10, 1.48)	.002
Depth of extension	Superficial	Reference	
	Deep	1.18 (1.07, 1.31)	<.001
	Unknown	1.01 (0.86, 1.19)	.863
Year of diagnosis	2004‐2005	Reference	
	2006‐2007	0.97 (0.87, 1.08)	.556
	2008‐2009	1.01 (0.90, 1.13)	.906
	2010‐2011	0.93 (0.81, 1.07)	.303
	2012‐2013	0.96 (0.82, 1.12)	.623
Chemotherapy	Not given	Reference	
	Given	0.91 (0.83, 1.01)	.069
	Unknown	0.64 (0.51, 0.81)	<.001
Surgery margin	Negative	Reference	
	Positive but unspecified extent	1.37 (1.17, 1.61)	<.001
	Microscopic residual	1.24 (1.10, 1.40)	<.001
	Macroscopic residual	2.01 (1.40, 2.89)	<.001
	Unknown	1.45 (1.22, 1.73)	<.001
Facility volume	≤7 (lowest 25%)	Reference	
	8‐81 (middle 50%)	0.87 (0.78, 0.97)	.015
	>81 (highest 25%)	0.75 (0.65, 0.86)	<.001

NE, not estimable; HR, hazard ratio; CI, confidence interval; LSS, limb‐sparing surgery; RT, radiation therapy; pre‐RT, preoperative RT; post‐RT, postoperative RT.

No significant interaction between treatment and tumor size.

**Table 2:** Multivariate analysis of survival using Surveillance, Epidemiology, and End Results Program

Variable	Category	Overall survival		Sarcoma mortality	
		HR (95% CI)	*P*‐value^a^	HR (95% CI)	*P*‐value^b^
Treatment	LSS	Reference		Reference	
	Amputation	1.59 (1.24, 2.04)	<.001	1.52 (1.12, 2.07)	.007
	Pre‐RT + LSS	0.60 (0.48, 0.75)	<.001	0.75 (0.58, 0.97)	.031
	LSS + post‐RT	0.70 (0.60, 0.82)	<.001	0.82 (0.67, 0.99)	.046
Facility location	Northeast	Reference		Reference	
	South	0.78 (0.60, 1.00)	.054	0.66 (0.49, 0.90)	.008
	Midwest	0.65 (0.49, 0.87)	.003	0.62 (0.44, 0.86)	.005
	West	0.77 (0.63, 0.94)	.012	0.75 (0.60, 0.94)	.013
Age at diagnosis	≤40	Reference		Reference	
	41‐50	1.10 (0.84, 1.43)	.504	1.05 (0.79, 1.40)	.745
	51‐60	1.07 (0.82, 1.39)	.618	0.88 (0.66, 1.16)	.365
	61‐70	1.28 (0.99, 1.66)	.061	0.93 (0.69, 1.23)	.595
	>70	2.82 (2.21, 3.59)	<.001	1.36 (1.04, 1.79)	.027
Sex	Male	Reference		Reference	
	Female	0.82 (0.71, 0.94)	.005	0.83 (0.70, 0.98)	.032
Marital status	Single	Reference		Reference	
	Married (including common law)/Domestic partner	0.64 (0.53, 0.78)	<.001	0.79 (0.63, 0.99)	.042
	Separated/Divorced	0.93 (0.72, 1.22)	.615	1.12 (0.82, 1.52)	.486
	Widowed	1.02 (0.79, 1.31)	.885	1.05 (0.76, 1.45)	.772
	Unknown	0.73 (0.46, 1.16)	.178	0.83 (0.47, 1.48)	.538
Race/ethnicity	White Non‐Hispanic	Reference		Reference	
	White Hispanic	1.09 (0.87, 1.35)	.466	1.14 (0.88, 1.47)	.315
	Black	0.97 (0.77, 1.22)	.789	1.14 (0.88, 1.48)	.315
	Other/Unknown	1.27 (1.00, 1.62)	.046	1.35 (1.03, 1.76)	.027
Median income	≥$63 000	Reference		Reference	
	$48 000‐$62 999	1.09 (0.92, 1.29)	.321	1.20 (0.99, 1.46)	.068
	$38 000‐$47 999	1.46 (1.12, 1.90)	.005	1.25 (0.91, 1.72)	.162
	<$38 000	1.88 (1.17, 3.01)	.009	1.76 (0.96, 3.23)	.069
Educational: Non‐High school	<7%	Reference		Reference	
	7%‐12.9%	1.13 (0.81, 1.59)	.465	1.04 (0.71, 1.51)	.844
	13%‐20.9%	1.09 (0.77, 1.54)	.641	1.08 (0.73, 1.59)	.700
	≥21%	1.17 (0.80, 1.72)	.421	0.92 (0.60, 1.43)	.725
Living location	Metro area	Reference		Reference	
	Small metro area	1.06 (0.90, 1.25)	.482	1.15 (0.95, 1.40)	.158
	Urban area	0.73 (0.54, 1.00)	.048	0.78 (0.52, 1.17)	.231
	Rural area	0.59 (0.28, 1.24)	.162	0.91 (0.38, 2.18)	.840
	Unknown	0.68 (0.09, 4.95)		NE	
Year of diagnosis	2004‐2005	Reference		Reference	
	2006‐2007	1.13 (0.94, 1.36)	.181	1.05 (0.85, 1.29)	.682
	2008‐2009	1.05 (0.86, 1.27)	.642	0.82 (0.66, 1.03)	.085
	2010‐2011	1.01 (0.82, 1.26)	.893	0.89 (0.70, 1.14)	.365
	2012‐2013	1.09 (0.79, 1.50)	.609	0.89 (0.62, 1.29)	.541
Primary tumor site	Upper limb	Reference		Reference	
	Lower limb	1.08 (0.92, 1.27)	.357	1.06 (0.86, 1.29)	.597
Tumor size (cm)	≤5	Reference		Reference	
	5.01‐10	1.66 (1.37, 2.02)	<.001	2.01 (1.57, 2.56)	<.001
	10.01‐15	2.71 (2.18, 3.36)	<.001	2.69 (2.04, 3.54)	<.001
	>15	3.66 (2.93, 4.58)	<.001	3.85 (2.92, 5.09)	<.001
Depth of extension	Superficial	Reference		Reference	
	Deep	1.21 (1.02, 1.43)	.025	1.41 (1.14, 1.74)	.001
	Unknown	1.12 (0.82, 1.54)	.460	1.27 (0.86, 1.88)	.233

HR, hazard ratio; CI, confidence interval; LSS, limb‐sparing surgery; pre‐RT, preoperative RT; post‐RT, postoperative RT.

a *P* values from multivariable Cox model.

b *P* value from Fine‐Gray sub‐distribution hazard model with mortality from other cause as competing risk.

Other selected factors associated with superior survival on MVA of the NCDB (Table 1) included younger age, female gender, private insurance, residence in an area with more high‐school graduates, fewer comorbidities, smaller tumor size, superficial tumor location, negative surgical margins, and treatment at a high‐volume center.

### 3.2 Propensity matched cohorts

Results of propensity score matching comparing LSS alone and preoperative RT with LSS are shown in Tables S3 (NCDB) and S4 (SEER). Most variables were significantly different between patients in each treatment group at baseline before matching. However, after propensity score matching, no variables remained significantly different between treatment groups, indicating successful matching. OS was improved with preoperative RT with LSS compared to LSS alone (Table S5) in both the NCDB (HR, 0.67; 95% CI: 0.58‐0.78) and SEER (HR, 0.57; 95% CI: 0.43‐0.75) MVAs. Preoperative RT was also associated with reduced SM (HR, 0.69; 95% CI: 0.49‐0.95). Median survival in the matched NCDB cohorts (Table 3) was 8.9 years (95% CI: 7.9 years‐not estimable) for preoperative RT and LSS vs 6.6 years for LSS alone (95% CI: 5.4‐7.8 years).

**Table 3:** Median survival and 3‐, 5‐, and 7‐y overall survival (OS) rates (%) using after propensity score matched data

Dataset	Treatment	Median survival (y)	OS rates (95% CI)		
			3 y	5 y	7 y
NCDB	LSS alone	6.6 (5.4, 7.8)	65.0 (61.9, 68.0)	55.0 (51.4, 58.3)	49.1 (45.1, 53.0)
	Pre‐RT + LSS	8.9 (7.9, NE)	73.9 (71.0, 76.6)	62.2 (58.8, 65.5)	56.1 (52.3, 59.8)
NCDB	LSS alone	7.2 (6.5, 8.9)	68.9 (66.6, 71.0)	58.6 (56.0, 61.0)	51.0 (48.1, 53.8)
	LSS + post‐RT	9.8 (9.0, 11.2)	76.6 (74.6, 78.5)	67.2 (64.8, 69.4)	59.2 (56.4, 61.8)
SEER	LSS alone	6.9 (4.7, NE)	64.3 (58.2, 69.9)	53.3 (46.2, 59.9)	48.2 (40.0, 55.8)
	Pre‐RT + LSS	9.2 (8.2, NE)	74.6 (69.0, 79.4)	65.4 (58.9, 71.1)	61.0 (53.8, 67.4)
SEER	LSS alone	8.1 (7.0, NE)	70.9 (67.0, 74.3)	60.9 (56.5, 65.0)	54.1 (48.9, 59.0)
	LSS + post‐RT	9.7 (8.8, NE)	79.4 (76.0, 82.4)	69.2 (65.1, 73.0)	62.1 (57.3, 66.5)

Dataset	Treatment	Cumulative incidence rates of sarcoma mortality (95% CI)		
		3 y	5 y	7 y
SEER	LSS alone	27.4 (22.1, 32.9)	35.2 (28.7, 41.6)	36.2 (29.6, 42.9)
	Pre‐RT + LSS	21.8 (17.1, 26.9)	28.7 (23.1, 34.5)	29.8 (23.9, 35.8)
SEER	LSS alone	19.6 (16.5, 22.8)	26.7 (23.0, 30.6)	29.9 (25.7, 34.3)
	LSS + post‐RT	16.2 (13.4, 19.3)	24.6 (21.0, 28.4)	26.3 (22.5, 30.3)

NE, not estimable; CI, confidence interval; LSS, limb‐sparing surgery; RT, radiation therapy; pre‐RT, preoperative RT; post‐RT, postoperative RT.

**Figure 2:**
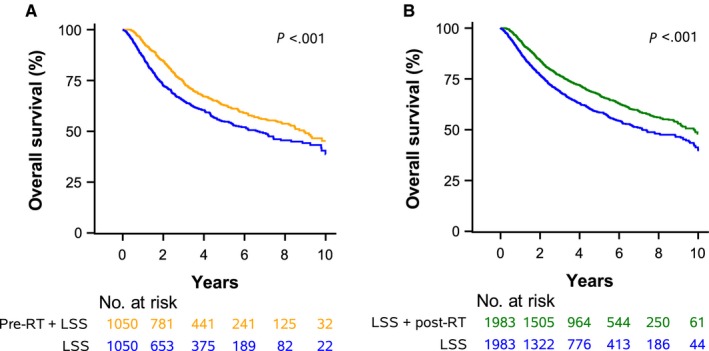
A. Preoperative Radiotherapy (pre‐RT) + Limb‐Sparing Surgery (LSS) vs. LSS alone. B. LSS + Postoperative Radiotherapy (post‐RT) vs. LSS alone

**Figure 3:**
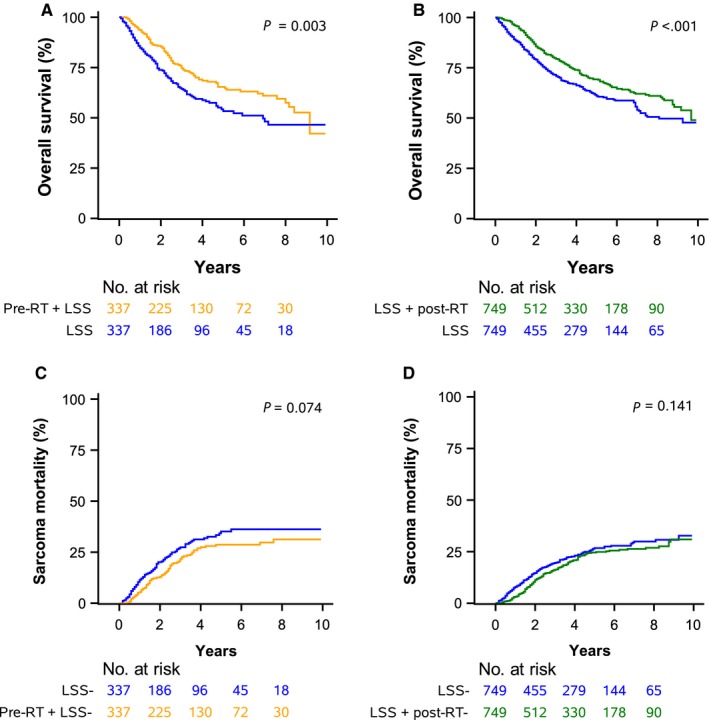
A. Overall Survival Comparison of Preoperative Radiotherapy (pre‐RT) + Limb‐Sparing Surgery (LSS) vs. LSS alone. B. Overall Survival Comparison of LSS + Postoperative Radiotherapy (post‐RT) vs. LSS alone. C. Sarcoma Mortality Comparison of pre‐RT + LSS vs. LSS alone. D. Sarcoma Mortality Comparison of LSS + post‐RT vs. LSS alone

When comparing LSS alone to LSS followed by postoperative RT, again, the cohorts were well matched after propensity score matching (Tables S6 and S7). LSS with postoperative RT was associated with superior survival in MVA in both NCDB (HR, 0.71; 95% CI: 0.64‐0.78) and SEER (HR, 0.71; 95% CI: 0.59‐0.86) data (Table S5). The difference in SM between patients receiving LSS with or without postoperative RT was not statistically significant (HR, 0.86; 95% CI: 0.68‐1.08). Using NCDB data (Table 3), LSS and postoperative RT resulted in a median survival of 9.8 years (95% CI: 9.0‐11.2) compared to 7.2 years (95% CI: 6.5‐8.9 years) for LSS alone. Survival outcomes are displayed for NCDB and SEER data in Figures 2 and 3.

## 4 DISCUSSION

In this first sarcoma analysis utilizing both the NCDB and SEER, modern data indicate that 28% (SEER) to 30% (NCDB) of patients with high‐grade E‐STS do not receive RT as part of a LSS approach. This analysis consistently showed that RT given before or after LSS resulted in significantly superior OS and SM compared to LSS alone. The results were consistent across both databases and with two different analysis techniques to adjust for baseline characteristics. Furthermore, patients treated with amputation alone consistently had significantly inferior OS and SM when compared to LSS approaches. No significant differences were noted in OS or SM when comparing preoperative and postoperative RT combined with LSS. Overall, the results indicate that LSS with either preoperative or postoperative RT should be the treatment of choice.

Previous randomized trials investigating local treatment modalities for E‐STS had very small sample sizes, ranging from 43 to 180 patients and thus were inadequately powered to detect small‐to‐moderate differences in OS between treatment arms.^3^, ^5^, ^6^, ^27^ For instance, one long‐term report of a trial comparing LSS alone to LSS with postoperative external beam RT found that 10‐ and 20‐year survival rates were 5% and 7% higher with the addition of RT, respectively. However, due to the small study size, these differences did not reach statistical significance.^26^ While the initial results of the trial on preoperative RT vs postoperative RT showed increased survival in the preoperative RT group,^6^ this survival benefit was not seen on longer follow.^28^ However, this study was designed with a primary endpoint of major wound complications and was not powered to assess OS differences.

Several large cancer registry studies have compared LSS alone to LSS combined with RT for patients with high‐grade E‐STS. In a SEER analysis of 983 patients with high‐grade E‐STS, 3‐year OS and disease‐specific survival were significantly superior with the addition of RT only in the subset of patients with tumors >5 cm.^12^ Another SEER analysis including 2689 patients with high‐grade E‐STS found significantly greater 3‐year OS in those who received RT (73% vs 63%). The survival difference was even more pronounced for patients with high‐grade tumors >5 cm in size (66% vs 53%); however, survival for smaller high‐grade tumors was not specified.^10^ Al‐Refaie et al^13^ published a SEER study of 1618 patients with E‐STSs <5 cm and found that adjuvant radiation did not significantly impact OS for low‐ or high‐grade disease, with 5‐year OS of 78.5% vs 76.8% in the high‐grade cohort. However, on MVA, there was a sizeable trend toward survival benefit (HR 0.67) of RT on OS that was minimally short of statistical significance (*P* = .071).^13^ An NCDB study included 10 290 patients with high‐grade E‐STS treated with LSS alone or LSS and RT from 1998 to 2006. After propensity score matching, 5‐year OS was 52% with RT vs 41% with no RT. Of note, this study did not distinguish between preoperative and postoperative RT; furthermore, unlike the current study, this prior NCDB study could not assess SM due to lack of information on this endpoint in the NCDB.^8^ Another NCDB study also showed a benefit to either preoperative or postoperative RT combined with resection although type of surgery (LSS or amputation) was not analyzed.^9^ Several other studies have shown a survival benefit to combine surgery and RT compared to surgery alone; however, they did not distinguish between LSS and amputation and also included patients with truncal sarcomas.^15^, ^29^

Our study, consistent with the results of the two larger registry studies described above,^8^, ^10^ showed an improvement in OS and SM with the addition of RT to LSS. While two of the above studies did not find a statistically significant benefit with radiation for patients with tumors <5 cm, survival rates were numerically higher with radiation in both arms, and neither was designed to test non‐inferiority. Furthermore, two randomized trials assessing the impact of adding postoperative RT to LSS showed a statistically significant reduction in local recurrences, and both trials included >40% of patients with tumors <5 cm.^5^, ^27^ In our study, tests for interaction showed no significant interaction between effect of RT and tumor size (data not shown).

Several studies have compared outcomes between preoperative and postoperative RT combined with LSS, and at least one has assessed the effect of amputation on survival. No significant difference in OS was seen between pre‐RT vs post‐RT in two SEER studies.^10^, ^11^ An NCDB study showed improved rates of negative surgical margins in patients receiving preoperative RT compared to postoperative RT; however, survival between these two treatment strategies was not directly compared.^9^ In contrast, a study of the National Oncology Database showed a significant OS and cause‐specific survival benefit to preoperative RT compared to postoperative RT. However, this study allowed stage I‐IV patients and included sarcomas of all sites rather than limiting the analysis to E‐STS.^18^ Our study, as in the above studies limited to E‐STS, showed no significant difference in outcomes when comparing preoperative RT and postoperative RT. This coincides with data from the randomized trial on this topic.^28^ Consistent with the results of our study, a previous SEER study of 6215 patients showed significantly worse SM for patients treated with amputation rather than LSS, an effect maintained in patients surviving 3 and 5 years.^14^

Multivariable analyses of the NCDB and SEER cohorts consistently found several additional variables associated with reduced OS including age >70, male gender, higher grade tumors, tumor size >5 cm, and deep extension, similar to the results of several other database studies.^10^, ^12^, ^14^, ^30^ The NCDB analysis also showed that treatment at a high‐volume center was associated with improved survival, consistent with previous analyses.^31^ Others have also found that postoperative RT, malignant fibrous histiocytoma, and liposarcoma histologies were predictors of improved OS.^11^, ^12^ While at least one SEER study^32^ found that black patients had worse SM and OS compared to white patients, our study did not find a similar difference based on race although this was not the primary variable evaluated in the current study. Our study identified additional variables associated with reduced OS including not having private insurance, living in communities with less high school graduates, and having more pre‐existing comorbidities. Of note, there was no significant difference in OS based on race nor ethnicity on MVA in either database.

There are several limitations to our analysis. Perhaps most notably is the retrospective nature of the study, which allows for the possibility of selection bias. While we attempted to adjust for differences in baseline characteristics via MVAs (both in unmatched and matched cohorts), unaccounted for variables could still have influenced treatment selection, potentially leading to misattribution of a survival benefit to treatment received. For patients receiving amputation, this is of particular concern given the increased morbidity associated with the procedure and the strong recommendation of national guidelines that this procedure be limited to select situations.^2^ The highly selected nature of this group is reflected in the small proportion of all patients (6.1% in the NCDB and 5.7% in SEER) and in the generally unfavorable baseline characteristics seen in patients receiving amputation. Therefore, certain variables which are not accounted for in these databases may have influenced a surgeon's decision to proceed with an amputation, and these unaccounted for variables, rather than the treatment itself, may have contributed to the difference in survival in this group. Immortal time bias^33^, ^34^ is a potential source of error in retrospective studies where a subject has to remain event free (ie, survive in the case of this analysis) to receive further treatment. This could be of particular concern in assessing the potential benefits of postoperative RT. Therefore, sensitivity analyses were conducted (data not shown) in patients surviving at least 1 year from diagnosis. Results were in the same direction in all cases.

While the use of chemotherapy was adjusted for in NCDB analyses, the specific chemotherapy regimens used are not reported to NCDB, and chemotherapy information was not available at all in the SEER dataset. Neither radiation dose nor histology type was included as covariates, and both could potentially impact survival outcomes. The insurance field in the SEER program does not record Medicare data; thus, many of the patients 65 and older with unknown insurance status likely had Medicare. A full 30.5% of our SEER sample had insurance status of “unknown.” Recently, significant concerns have been expressed about the accuracy of the radiation variable in SEER with rates of RT likely underreported;^35^ however, given the consistent benefits to RT seen in our analysis of both databases, this lack of reporting would likely lead to dilution of RT's true benefit in SEER rather than its overestimation. Furthermore, neither database provides information on important endpoints including LC, distant metastases, physician‐reported adverse effects, or patient‐reported outcomes. Important patient and treatment variables such as the comorbidity status, the presence of positive margins, RT details, and systemic therapy information are not provided in the SEER program.

## 5 CONCLUSION

Many patients still receive amputation or LSS without RT. Our study found that adding RT (either preoperative or postoperatively) to LSS was associated with increased OS and reduced SM in patients with high‐grade E‐STS. Amputation was associated with worse survival outcomes in all analyses. There were no significant differences in survival based on the timing of radiotherapy (ie, preoperative or postoperative). In conclusion, LSS combined with RT is the optimal treatment option for most patients with high‐grade E‐STS.

## CONFLICTS OF INTEREST

Stephen Ramey: Travel Accommodations from Targeted Therapies. Breelyn Wilky: Consulting or Advisory Role for Novartis, Janssen Oncology, and Lilly; Research Funding from Novartis, Merck Sharp & Dohme, Daiichi Sankyo, ArQule, Agenus; Travel Accommodations from Novartis, Lilly, Advenchen Laboratories. Jonathan Trent: Honoraria from GlaxoSmithKline; Consulting or Advisory Role from Novartis, Lilly, Janssen. All other authors report no disclosures.
