# Supplementary material for: Limb‐sparing surgery plus radiotherapy results in superior survival: an analysis of patients with high‐grade, extremity soft‐tissue sarcoma from the NCDB and SEER
Source: Cancer Med. 2018 Jul 20;7(9):4228–39. doi: 10.1002/cam4.1625 (PMC6144142; doi:10.1002/cam4.1625)
Supplement: Supplementary file 3 [file CAM4-7-4228-s003.docx]

| **Supplemental Table 1.** Demographics and clinical characteristics of included high-grade extremity sarcoma patients by treatment in the National Cancer Database (NCDB) | | | | | | | | | | | | | | | | |
| --- | --- | --- | --- | --- | --- | --- | --- | --- | --- | --- | --- | --- | --- | --- | --- | --- |
| Variable | | Category | | Total | | Treatment | | | | | | | | |  | |
|  |  |  |  |  |  | Amputation | | | LSS | | Pre-RT + LSS | | LSS + post-RT | |  | |
|  |  |  |  | N | % | N | | % | N | % | N | % | N | % | P^1^ | |
| Total | |  | 7,828 | 100.0 | | 477 | 100.0 | 2,203 | 100.0 | 1,589 | 100.0 | 3,559 | 100.0 | |  |  |
| Facility type | | Community Program | 2,177 | 27.8 | | 83 | 17.4 | 582 | 26.4 | 318 | 20.0 | 1,194 | 33.5 | | <.001 |  |
|  | | Academic/Research Program | 3,740 | 47.8 | | 248 | 52.0 | 1,060 | 48.1 | 837 | 52.7 | 1,595 | 44.8 | |  |  |
|  | | Integrated Network Cancer Program | 860 | 11.0 | | 50 | 10.5 | 230 | 10.4 | 240 | 15.1 | 340 | 9.6 | |  |  |
|  | | Other/Unknown | 1,051 | 13.4 | | 96 | 20.1 | 331 | 15.0 | 194 | 12.2 | 430 | 12.1 | |  |  |
| Facility location | | Northeast | 1,409 | 18.0 | | 77 | 16.1 | 424 | 19.2 | 247 | 15.5 | 661 | 18.6 | | <.001 |  |
|  | | South | 2,377 | 30.4 | | 142 | 29.8 | 685 | 31.1 | 599 | 37.7 | 951 | 26.7 | |  |  |
|  | | Midwest | 1,710 | 21.8 | | 96 | 20.1 | 388 | 17.6 | 366 | 23.0 | 860 | 24.2 | |  |  |
|  | | West | 1,281 | 16.4 | | 66 | 13.8 | 375 | 17.0 | 183 | 11.5 | 657 | 18.5 | |  |  |
|  | | Other/Unknown | 1,051 | 13.4 | | 96 | 20.1 | 331 | 15.0 | 194 | 12.2 | 430 | 12.1 | |  |  |
| Age in years | | ≤40 | 1,114 | 14.2 | | 100 | 21.0 | 346 | 15.7 | 202 | 12.7 | 466 | 13.1 | | <.001 |  |
|  | | 41-50 | 1,084 | 13.8 | | 62 | 13.0 | 278 | 12.6 | 234 | 14.7 | 510 | 14.3 | |  |  |
|  | | 51-60 | 1,616 | 20.6 | | 93 | 19.5 | 395 | 17.9 | 371 | 23.3 | 757 | 21.3 | |  |  |
|  | | 61-70 | 1,597 | 20.4 | | 89 | 18.7 | 403 | 18.3 | 380 | 23.9 | 725 | 20.4 | |  |  |
|  | | >70 | 2,417 | 30.9 | | 133 | 27.9 | 781 | 35.5 | 402 | 25.3 | 1,101 | 30.9 | |  |  |
| Sex | | Male | 4,183 | 53.4 | | 267 | 56.0 | 1,133 | 51.4 | 871 | 54.8 | 1,912 | 53.7 | | 0.110 |  |
|  | | Female | 3,645 | 46.6 | | 210 | 44.0 | 1,070 | 48.6 | 718 | 45.2 | 1,647 | 46.3 | |  |  |
| Race/ethnicity | | White Non-Hispanic | 5,794 | 74.0 | | 329 | 69.0 | 1,604 | 72.8 | 1,226 | 77.2 | 2,635 | 74.0 | | <.001 |  |
|  | | White Hispanic | 476 | 6.1 | | 42 | 8.8 | 157 | 7.1 | 65 | 4.1 | 212 | 6.0 | |  |  |
|  | | Black | 802 | 10.2 | | 65 | 13.6 | 213 | 9.7 | 184 | 11.6 | 340 | 9.6 | |  |  |
|  | | Other/Unknown | 756 | 9.7 | | 41 | 8.6 | 229 | 10.4 | 114 | 7.2 | 372 | 10.5 | |  |  |
| Insurance status | | Private Insurance | 3,740 | 47.8 | | 196 | 41.1 | 935 | 42.4 | 831 | 52.3 | 1,778 | 50.0 | | <.001 |  |
|  | | Not Insured | 318 | 4.1 | | 33 | 6.9 | 109 | 4.9 | 55 | 3.5 | 121 | 3.4 | |  |  |
|  | | Medicaid | 446 | 5.7 | | 52 | 10.9 | 130 | 5.9 | 74 | 4.7 | 190 | 5.3 | |  |  |
|  | | Medicare | 3,078 | 39.3 | | 186 | 39.0 | 965 | 43.8 | 556 | 35.0 | 1,371 | 38.5 | |  |  |
|  | | Other/Unknown | 246 | 3.1 | | 10 | 2.1 | 64 | 2.9 | 73 | 4.6 | 99 | 2.8 | |  |  |
| Median income 2012 | | ≥$63000 | 2,568 | 32.8 | | 123 | 25.8 | 699 | 31.7 | 527 | 33.2 | 1,219 | 34.3 | | 0.002 |  |
|  | | $48,000-$62,999 | 2,055 | 26.3 | | 116 | 24.3 | 575 | 26.1 | 409 | 25.7 | 955 | 26.8 | |  |  |
|  | | $38,000-$47,999 | 1,790 | 22.9 | | 125 | 26.2 | 501 | 22.7 | 358 | 22.5 | 806 | 22.6 | |  |  |
|  | | <$38,000 | 1,282 | 16.4 | | 103 | 21.6 | 388 | 17.6 | 266 | 16.7 | 525 | 14.8 | |  |  |
|  | | Unknown | 133 | 1.7 | | 10 | 2.1 | 40 | 1.8 | 29 | 1.8 | 54 | 1.5 | |  |  |
| Percent non-High school | | <7% | 2,017 | 25.8 | | 92 | 19.3 | 548 | 24.9 | 422 | 26.6 | 955 | 26.8 | | <.001 |  |
|  | | 7-12.9% | 2,534 | 32.4 | | 141 | 29.6 | 722 | 32.8 | 503 | 31.7 | 1,168 | 32.8 | |  |  |
|  | | 13-20.9% | 1,879 | 24.0 | | 125 | 26.2 | 513 | 23.3 | 404 | 25.4 | 837 | 23.5 | |  |  |
|  | | ≥21% | 1,278 | 16.3 | | 109 | 22.9 | 384 | 17.4 | 234 | 14.7 | 551 | 15.5 | |  |  |
|  | | Unknown | 120 | 1.5 | | 10 | 2.1 | 36 | 1.6 | 26 | 1.6 | 48 | 1.3 | |  |  |
| Living location | | Metro area | 3,913 | 50.0 | | 197 | 41.3 | 1,110 | 50.4 | 754 | 47.5 | 1,852 | 52.0 | | 0.002 |  |
|  | | Smaller metro area | 2,373 | 30.3 | | 165 | 34.6 | 675 | 30.6 | 496 | 31.2 | 1,037 | 29.1 | |  |  |
|  | | Urban area | 1,147 | 14.7 | | 88 | 18.4 | 306 | 13.9 | 246 | 15.5 | 507 | 14.2 | |  |  |
|  | | Rural area | 110 | 1.4 | | 9 | 1.9 | 30 | 1.4 | 31 | 2.0 | 40 | 1.1 | |  |  |
|  | | Unknown | 285 | 3.6 | | 18 | 3.8 | 82 | 3.7 | 62 | 3.9 | 123 | 3.5 | |  |  |
| Distance to hospital | | ≤10 | 2,709 | 34.6 | | 113 | 23.7 | 678 | 30.8 | 415 | 26.1 | 1,503 | 42.2 | | <.001 |  |
|  | | 11-20 | 1,447 | 18.5 | | 81 | 17.0 | 402 | 18.2 | 276 | 17.4 | 688 | 19.3 | |  |  |
|  | | 21-50 | 1,565 | 20.0 | | 104 | 21.8 | 420 | 19.1 | 348 | 21.9 | 693 | 19.5 | |  |  |
|  | | >50 | 1,989 | 25.4 | | 170 | 35.6 | 667 | 30.3 | 524 | 33.0 | 628 | 17.6 | |  |  |
|  | | Unknown | 118 | 1.5 | | 9 | 1.9 | 36 | 1.6 | 26 | 1.6 | 47 | 1.3 | |  |  |
| Comorbidity | | 0 | 6,423 | 82.1 | | 368 | 77.1 | 1,770 | 80.3 | 1,298 | 81.7 | 2,987 | 83.9 | | 0.001 |  |
|  | | 1 | 1,155 | 14.8 | | 88 | 18.4 | 351 | 15.9 | 242 | 15.2 | 474 | 13.3 | |  |  |
|  | | 2 | 250 | 3.2 | | 21 | 4.4 | 82 | 3.7 | 49 | 3.1 | 98 | 2.8 | |  |  |
| Transition in care | | No | 1,045 | 13.3 | | 67 | 14.0 | 332 | 15.1 | 239 | 15.0 | 407 | 11.4 | | <.001 |  |
|  | | Yes | 4,227 | 54.0 | | 252 | 52.8 | 1,076 | 48.8 | 886 | 55.8 | 2,013 | 56.6 | |  |  |
|  | | Unknown | 2,556 | 32.7 | | 158 | 33.1 | 795 | 36.1 | 464 | 29.2 | 1,139 | 32.0 | |  |  |
| Date of diagnosis | | 2004-2005 | 1,372 | 17.5 | | 87 | 18.2 | 400 | 18.2 | 220 | 13.8 | 665 | 18.7 | | <.001 |  |
|  | | 2006-2007 | 1,522 | 19.4 | | 99 | 20.8 | 414 | 18.8 | 257 | 16.2 | 752 | 21.1 | |  |  |
|  | | 2008-2009 | 1,684 | 21.5 | | 101 | 21.2 | 499 | 22.7 | 326 | 20.5 | 758 | 21.3 | |  |  |
|  | | 2010-2011 | 1,609 | 20.6 | | 103 | 21.6 | 441 | 20.0 | 362 | 22.8 | 703 | 19.8 | |  |  |
|  | | 2012-2013 | 1,641 | 21.0 | | 87 | 18.2 | 449 | 20.4 | 424 | 26.7 | 681 | 19.1 | |  |  |
| Primary site | | Upper limb | 1,891 | 24.2 | | 98 | 20.5 | 541 | 24.6 | 330 | 20.8 | 922 | 25.9 | | <.001 |  |
|  | | Lower limb | 5,937 | 75.8 | | 379 | 79.5 | 1,662 | 75.4 | 1,259 | 79.2 | 2,637 | 74.1 | |  |  |
| Grade | | Poorly differentiated | 4,698 | 60.0 | | 274 | 57.4 | 1,340 | 60.8 | 884 | 55.6 | 2,200 | 61.8 | | <.001 |  |
|  | | Undifferentiated; anaplastic | 3,130 | 40.0 | | 203 | 42.6 | 863 | 39.2 | 705 | 44.4 | 1,359 | 38.2 | |  |  |
| Tumor size (cm) | | ≤5 | 2,213 | 28.3 | | 72 | 15.1 | 846 | 38.4 | 206 | 13.0 | 1,089 | 30.6 | | <.001 |  |
|  | | 5.01-10 | 2,914 | 37.2 | | 168 | 35.2 | 743 | 33.7 | 630 | 39.6 | 1,373 | 38.6 | |  |  |
|  | | 10.01-15 | 1,498 | 19.1 | | 110 | 23.1 | 325 | 14.8 | 401 | 25.2 | 662 | 18.6 | |  |  |
|  | | >15 | 1,203 | 15.4 | | 127 | 26.6 | 289 | 13.1 | 352 | 22.2 | 435 | 12.2 | |  |  |
| Clinical tumor stage | | I | 1,628 | 20.8 | | 51 | 10.7 | 633 | 28.7 | 158 | 9.9 | 786 | 22.1 | | <.001 |  |
|  | | II | 4,359 | 55.7 | | 298 | 62.5 | 1,019 | 46.3 | 1,271 | 80.0 | 1,771 | 49.8 | |  |  |
|  | | Unknown | 1,841 | 23.5 | | 128 | 26.8 | 551 | 25.0 | 160 | 10.1 | 1,002 | 28.2 | |  |  |
| Depth of extension | | Superficial | 1,962 | 25.1 | | 56 | 11.7 | 731 | 33.2 | 204 | 12.8 | 971 | 27.3 | | <.001 |  |
|  | | Deep | 5,232 | 66.8 | | 396 | 83.0 | 1,250 | 56.7 | 1,285 | 80.9 | 2,301 | 64.7 | |  |  |
|  | | Unknown | 634 | 8.1 | | 25 | 5.2 | 222 | 10.1 | 100 | 6.3 | 287 | 8.1 | |  |  |
| Chemotherapy | | None | 5,743 | 73.4 | | 302 | 63.3 | 1,762 | 80.0 | 1,008 | 63.4 | 2,671 | 75.0 | | <.001 |  |
|  | | Given | 1,862 | 23.8 | | 156 | 32.7 | 373 | 16.9 | 543 | 34.2 | 790 | 22.2 | |  |  |
|  | | Unknown | 223 | 2.8 | | 19 | 4.0 | 68 | 3.1 | 38 | 2.4 | 98 | 2.8 | |  |  |
| Surgery margin | | Negative | 6,458 | 82.5 | | 453 | 95.0 | 1,815 | 82.4 | 1,413 | 88.9 | 2,777 | 78.0 | | <.001 |  |
|  | | Positive but unspecified extent | 374 | 4.8 | | 8 | 1.7 | 106 | 4.8 | 41 | 2.6 | 219 | 6.2 | |  |  |
|  | | Microscopic residual | 652 | 8.3 | | 5 | 1.0 | 186 | 8.4 | 67 | 4.2 | 394 | 11.1 | |  |  |
|  | | Macroscopic residual | 47 | 0.6 | | - | - | 12 | 0.5 | 5 | 0.3 | 30 | 0.8 | |  |  |
|  | | Unknown | 297 | 3.8 | | 11 | 2.3 | 84 | 3.8 | 63 | 4.0 | 139 | 3.9 | |  |  |
| Facility volume | | ≤7 (Lowest 25%) | 1,976 | 25.2 | | 63 | 13.2 | 522 | 23.7 | 246 | 15.5 | 1,145 | 32.2 | | <.001 |  |
|  | | 8-81 (Middle 50% | 3,930 | 50.2 | | 259 | 54.3 | 1,172 | 53.2 | 836 | 52.6 | 1,663 | 46.7 | |  |  |
|  | | >81 (Highest 25%) | 1,922 | 24.6 | | 155 | 32.5 | 509 | 23.1 | 507 | 31.9 | 751 | 21.1 | |  |  |
| Abbreviations: Limb-sparing surgery (LSS), radiotherapy (RT), pre-operative RT (pre-RT), post-operative RT (post-RT), centimeters (cm).  ^1^P-values from chi-square test. | | | | | | | | | | | | | | | |  |

| **Supplemental Table 2.** Demographics and clinical characteristics of included high-grade extremity sarcoma patients  by treatment (limb-sparing surgery alone versus pre-operative radiotherapy + limb-sparing surgery) from the Surveillance, Epidemiology, and End Results (SEER) Program | | | | | | | | | | | |
| --- | --- | --- | --- | --- | --- | --- | --- | --- | --- | --- | --- |
|  |  | | Treatment | | | | | | | |  |
| Variable | Total | | Amputation | | LSS | | Pre-RT + LSS | | LSS + post-RT | |  |
|  | N | % | N | % | N | % | N | % | N | % | P^1^ |
| Total | 2,937 | 100.0 | 168 | 100.0 | 775 | 100.0 | 484 | 100.0 | 1,510 | 100.0 |  |
| Facility location |  |  |  |  |  |  |  |  |  |  |  |
| Northeast | 449 | 15.3 | 21 | 12.5 | 145 | 18.7 | 53 | 11.0 | 230 | 15.2 | <.001 |
| South | 612 | 20.8 | 37 | 22.0 | 157 | 20.3 | 184 | 38.0 | 234 | 15.5 |  |
| Midwest | 281 | 9.6 | 24 | 14.3 | 72 | 9.3 | 38 | 7.9 | 147 | 9.7 |  |
| West | 1,595 | 54.3 | 86 | 51.2 | 401 | 51.7 | 209 | 43.2 | 899 | 59.5 |  |
| Age in years |  |  |  |  |  |  |  |  |  |  |  |
| ≤40 | 427 | 14.5 | 31 | 18.5 | 116 | 15.0 | 67 | 13.8 | 213 | 14.1 | 0.038 |
| 41-50 | 456 | 15.5 | 26 | 15.5 | 118 | 15.2 | 76 | 15.7 | 236 | 15.6 |  |
| 51-60 | 592 | 20.2 | 30 | 17.9 | 143 | 18.5 | 112 | 23.1 | 307 | 20.3 |  |
| 61-70 | 587 | 20.0 | 33 | 19.6 | 141 | 18.2 | 117 | 24.2 | 296 | 19.6 |  |
| >70 | 875 | 29.8 | 48 | 28.6 | 257 | 33.2 | 112 | 23.1 | 458 | 30.3 |  |
| Sex |  |  |  |  |  |  |  |  |  |  |  |
| Male | 1,629 | 55.5 | 104 | 61.9 | 431 | 55.6 | 273 | 56.4 | 821 | 54.4 | 0.292 |
| Female | 1,308 | 44.5 | 64 | 38.1 | 344 | 44.4 | 211 | 43.6 | 689 | 45.6 |  |
| Marital status |  |  |  |  |  |  |  |  |  |  |  |
| Single (never married) | 530 | 18.0 | 34 | 20.2 | 142 | 18.3 | 83 | 17.1 | 271 | 17.9 | 0.058 |
| Married (including common law) /Domestic Partner | 1,724 | 58.7 | 97 | 57.7 | 418 | 53.9 | 301 | 62.2 | 908 | 60.1 |  |
| Separated/Divorced | 261 | 8.9 | 11 | 6.5 | 74 | 9.5 | 37 | 7.6 | 139 | 9.2 |  |
| Widowed | 336 | 11.4 | 21 | 12.5 | 109 | 14.1 | 53 | 11.0 | 153 | 10.1 |  |
| Unknown | 86 | 2.9 | 5 | 3.0 | 32 | 4.1 | 10 | 2.1 | 39 | 2.6 |  |
| Race/ethnicity |  |  |  |  |  |  |  |  |  |  |  |
| White Non-Hispanic | 2,023 | 68.9 | 99 | 58.9 | 529 | 68.3 | 347 | 71.7 | 1,048 | 69.4 | 0.005 |
| White Hispanic | 363 | 12.4 | 27 | 16.1 | 102 | 13.2 | 46 | 9.5 | 188 | 12.5 |  |
| Black | 286 | 9.7 | 27 | 16.1 | 68 | 8.8 | 58 | 12.0 | 133 | 8.8 |  |
| Other/Unknown | 265 | 9.0 | 15 | 8.9 | 76 | 9.8 | 33 | 6.8 | 141 | 9.3 |  |
| Insurance status |  |  |  |  |  |  |  |  |  |  |  |
| Not Insured | 89 | 3.0 | 9 | 5.4 | 33 | 4.3 | 16 | 3.3 | 31 | 2.1 | <.001 |
| Insured/no specifics | 1,772 | 60.3 | 87 | 51.8 | 450 | 58.1 | 330 | 68.2 | 905 | 59.9 |  |
| Medicaid | 180 | 6.1 | 21 | 12.5 | 57 | 7.4 | 24 | 5.0 | 78 | 5.2 |  |
| Other/Unknown | 896 | 30.5 | 51 | 30.4 | 235 | 30.3 | 114 | 23.6 | 496 | 32.8 |  |
| Median household income |  |  |  |  |  |  |  |  |  |  |  |
| $63,000 + | 1,204 | 41.0 | 55 | 32.7 | 296 | 38.2 | 168 | 34.7 | 685 | 45.4 | <.001 |
| $48,000-$62,999 | 1,164 | 39.6 | 71 | 42.3 | 324 | 41.8 | 201 | 41.5 | 568 | 37.6 |  |
| $38,000-$47,999 | 427 | 14.5 | 34 | 20.2 | 107 | 13.8 | 73 | 15.1 | 213 | 14.1 |  |
| <$38,000 | 142 | 4.8 | 8 | 4.8 | 48 | 6.2 | 42 | 8.7 | 44 | 2.9 |  |
| Education: Non-High school |  |  |  |  |  |  |  |  |  |  |  |
| <7% | 138 | 4.7 | 5 | 3.0 | 40 | 5.2 | 26 | 5.4 | 67 | 4.4 | 0.326 |
| 7-12.9% | 1,103 | 37.6 | 54 | 32.1 | 275 | 35.5 | 183 | 37.8 | 591 | 39.1 |  |
| 13-20.9% | 1,009 | 34.4 | 62 | 36.9 | 264 | 34.1 | 160 | 33.1 | 523 | 34.6 |  |
| ≥21% | 687 | 23.4 | 47 | 28.0 | 196 | 25.3 | 115 | 23.8 | 329 | 21.8 |  |
| Living location |  |  |  |  |  |  |  |  |  |  |  |
| Metro area | 1,907 | 64.9 | 99 | 58.9 | 491 | 63.4 | 292 | 60.3 | 1,025 | 67.9 | <.001 |
| Small metro area | 731 | 24.9 | 46 | 27.4 | 189 | 24.4 | 127 | 26.2 | 369 | 24.4 |  |
| Urban area | 270 | 9.2 | 20 | 11.9 | 86 | 11.1 | 59 | 12.2 | 105 | 7.0 |  |
| Rural area | 26 | 0.9 | 3 | 1.8 | 7 | 0.9 | 6 | 1.2 | 10 | 0.7 |  |
| Unknown | 3 | 0.1 | - | - | 2 | 0.3 | - | - | 1 | 0.1 |  |
| Date of diagnosis |  |  |  |  |  |  |  |  |  |  |  |
| 2004-2005 | 566 | 19.3 | 37 | 22.0 | 143 | 18.5 | 66 | 13.6 | 320 | 21.2 | 0.002 |
| 2006-2007 | 564 | 19.2 | 31 | 18.5 | 149 | 19.2 | 81 | 16.7 | 303 | 20.1 |  |
| 2008-2009 | 594 | 20.2 | 33 | 19.6 | 173 | 22.3 | 90 | 18.6 | 298 | 19.7 |  |
| 2010-2011 | 612 | 20.8 | 35 | 20.8 | 158 | 20.4 | 118 | 24.4 | 301 | 19.9 |  |
| 2012-2013 | 601 | 20.5 | 32 | 19.0 | 152 | 19.6 | 129 | 26.7 | 288 | 19.1 |  |
| Primary site |  |  |  |  |  |  |  |  |  |  |  |
| Upper limb | 717 | 24.4 | 36 | 21.4 | 204 | 26.3 | 103 | 21.3 | 374 | 24.8 | 0.170 |
| Lower limb | 2,220 | 75.6 | 132 | 78.6 | 571 | 73.7 | 381 | 78.7 | 1,136 | 75.2 |  |
| Grade |  |  |  |  |  |  |  |  |  |  |  |
| Poorly differentiated | 1,166 | 39.7 | 62 | 36.9 | 326 | 42.1 | 157 | 32.4 | 621 | 41.1 | 0.003 |
| Undifferentiated; anaplastic | 1,771 | 60.3 | 106 | 63.1 | 449 | 57.9 | 327 | 67.6 | 889 | 58.9 |  |
| Tumor size (cm) |  |  |  |  |  |  |  |  |  |  |  |
| ≤5 | 1,012 | 34.5 | 39 | 23.2 | 385 | 49.7 | 76 | 15.7 | 512 | 33.9 | <.001 |
| 5.01-10 | 983 | 33.5 | 49 | 29.2 | 221 | 28.5 | 178 | 36.8 | 535 | 35.4 |  |
| 10.01-15 | 529 | 18.0 | 34 | 20.2 | 95 | 12.3 | 130 | 26.9 | 270 | 17.9 |  |
| >15 | 413 | 14.1 | 46 | 27.4 | 74 | 9.5 | 100 | 20.7 | 193 | 12.8 |  |
| AJCC tumor stage |  |  |  |  |  |  |  |  |  |  |  |
| II | 1,420 | 48.3 | 60 | 35.7 | 491 | 63.4 | 129 | 26.7 | 740 | 49.0 | <.001 |
| III | 1,517 | 51.7 | 108 | 64.3 | 284 | 36.6 | 355 | 73.3 | 770 | 51.0 |  |
| Depth of extension |  |  |  |  |  |  |  |  |  |  |  |
| Superficial | 812 | 27.6 | 30 | 17.9 | 269 | 34.7 | 72 | 14.9 | 441 | 29.2 | <.001 |
| Deep | 1,910 | 65.0 | 131 | 78.0 | 407 | 52.5 | 401 | 82.9 | 971 | 64.3 |  |
| Unknown | 215 | 7.3 | 7 | 4.2 | 99 | 12.8 | 11 | 2.3 | 98 | 6.5 |  |
| Cause of death |  |  |  |  |  |  |  |  |  |  |  |
| Alive | 2,005 | 68.3 | 79 | 47.0 | 523 | 67.5 | 350 | 72.3 | 1,053 | 69.7 | <.001 |
| Death due to sarcoma | 671 | 22.8 | 69 | 41.1 | 164 | 21.2 | 104 | 21.5 | 334 | 22.1 |  |
| Death due to other causes | 261 | 8.9 | 20 | 11.9 | 88 | 11.4 | 30 | 6.2 | 123 | 8.1 |  |
| Abbreviations: Limb-sparing surgery (LSS), radiotherapy (RT), pre-operative RT (pre-RT), post-operative RT (post-RT), centimeters (cm), American Joint Committee on Cancer (AJCC).  ^1^P-value from chi-square test. | | | | | | | | | | | |
|  | | | | | | | | | | | |

| **Supplemental Table 3**. Demographics and clinical characteristics of included high-grade extremity sarcoma patients by treatment (limb-sparing surgery alone versus pre-operative radiotherapy + limb-sparing surgery) before and after 1:1 matching from the National Cancer Database (NCDB) | | | | | | | | | | | | | | | |
| --- | --- | --- | --- | --- | --- | --- | --- | --- | --- | --- | --- | --- | --- | --- | --- |
|  |  | Unmatched | | | | | | | 1:1 matched | | | | | | |
|  | Category | Total | | Treatment | | | |  | Total | | Treatment | | | |  |
| Variable |  |  |  | LSS | | Pre-RT + LSS | |  |  |  | LSS | | Pre-RT + LSS | |  |
|  |  | N | % | N | % | N | % | P^1^ | N | % | N | % | N | % | P^1^ |
| Total |  | 3,792 | 100.0 | 2,203 | 100.0 | 1,589 | 100.0 |  | 2,100 | 100.0 | 1,050 | 100.0 | 1,050 | 100.0 |  |
| Facility type | Community Program | 900 | 23.7 | 582 | 26.4 | 318 | 20.0 | <.001 | 455 | 21.7 | 231 | 22.0 | 224 | 21.3 | 0.816 |
|  | Academic/Research Program | 1,897 | 50.0 | 1,060 | 48.1 | 837 | 52.7 |  | 1,106 | 52.7 | 557 | 53.0 | 549 | 52.3 |  |
|  | Integrated Network Cancer Program | 470 | 12.4 | 230 | 10.4 | 240 | 15.1 |  | 248 | 11.8 | 124 | 11.8 | 124 | 11.8 |  |
|  | Other/Unknown | 525 | 13.8 | 331 | 15.0 | 194 | 12.2 |  | 291 | 13.9 | 138 | 13.1 | 153 | 14.6 |  |
| Facility location | Northeast | 671 | 17.7 | 424 | 19.2 | 247 | 15.5 | <.001 | 375 | 17.9 | 189 | 18.0 | 186 | 17.7 | 0.916 |
|  | South | 1,284 | 33.9 | 685 | 31.1 | 599 | 37.7 |  | 687 | 32.7 | 344 | 32.8 | 343 | 32.7 |  |
|  | Midwest | 754 | 19.9 | 388 | 17.6 | 366 | 23.0 |  | 444 | 21.1 | 225 | 21.4 | 219 | 20.9 |  |
|  | West | 558 | 14.7 | 375 | 17.0 | 183 | 11.5 |  | 303 | 14.4 | 154 | 14.7 | 149 | 14.2 |  |
|  | Other/Unknown | 525 | 13.8 | 331 | 15.0 | 194 | 12.2 |  | 291 | 13.9 | 138 | 13.1 | 153 | 14.6 |  |
| Age in years | ≤40 | 548 | 14.5 | 346 | 15.7 | 202 | 12.7 | <.001 | 300 | 14.3 | 142 | 13.5 | 158 | 15.0 | 0.890 |
|  | 41-50 | 512 | 13.5 | 278 | 12.6 | 234 | 14.7 |  | 296 | 14.1 | 152 | 14.5 | 144 | 13.7 |  |
|  | 51-60 | 766 | 20.2 | 395 | 17.9 | 371 | 23.3 |  | 432 | 20.6 | 216 | 20.6 | 216 | 20.6 |  |
|  | 61-70 | 783 | 20.6 | 403 | 18.3 | 380 | 23.9 |  | 427 | 20.3 | 215 | 20.5 | 212 | 20.2 |  |
|  | >70 | 1,183 | 31.2 | 781 | 35.5 | 402 | 25.3 |  | 645 | 30.7 | 325 | 31.0 | 320 | 30.5 |  |
| Sex | Male | 2,004 | 52.8 | 1,133 | 51.4 | 871 | 54.8 | 0.039 | 1,106 | 52.7 | 540 | 51.4 | 566 | 53.9 | 0.256 |
|  | Female | 1,788 | 47.2 | 1,070 | 48.6 | 718 | 45.2 |  | 994 | 47.3 | 510 | 48.6 | 484 | 46.1 |  |
| Race/ethnicity | White Non-Hispanic | 2,830 | 74.6 | 1,604 | 72.8 | 1,226 | 77.2 | <.001 | 1,576 | 75.0 | 784 | 74.7 | 792 | 75.4 | 0.931 |
|  | White Hispanic | 222 | 5.9 | 157 | 7.1 | 65 | 4.1 |  | 106 | 5.0 | 52 | 5.0 | 54 | 5.1 |  |
|  | Black | 397 | 10.5 | 213 | 9.7 | 184 | 11.6 |  | 225 | 10.7 | 117 | 11.1 | 108 | 10.3 |  |
|  | Other/Unknown | 343 | 9.0 | 229 | 10.4 | 114 | 7.2 |  | 193 | 9.2 | 97 | 9.2 | 96 | 9.1 |  |
| Insurance status | Private Insurance | 1,766 | 46.6 | 935 | 42.4 | 831 | 52.3 | <.001 | 985 | 46.9 | 488 | 46.5 | 497 | 47.3 | 0.984 |
|  | Not Insured | 164 | 4.3 | 109 | 4.9 | 55 | 3.5 |  | 93 | 4.4 | 45 | 4.3 | 48 | 4.6 |  |
|  | Medicaid | 204 | 5.4 | 130 | 5.9 | 74 | 4.7 |  | 115 | 5.5 | 58 | 5.5 | 57 | 5.4 |  |
|  | Medicare | 1,521 | 40.1 | 965 | 43.8 | 556 | 35.0 |  | 830 | 39.5 | 419 | 39.9 | 411 | 39.1 |  |
|  | Other/Unknown | 137 | 3.6 | 64 | 2.9 | 73 | 4.6 |  | 77 | 3.7 | 40 | 3.8 | 37 | 3.5 |  |
| Median income 2012 | ≥$63,000 | 1,226 | 32.3 | 699 | 31.7 | 527 | 33.2 | 0.787 | 678 | 32.3 | 338 | 32.2 | 340 | 32.4 | 0.999 |
|  | $48,000-$62,999 | 984 | 25.9 | 575 | 26.1 | 409 | 25.7 |  | 561 | 26.7 | 280 | 26.7 | 281 | 26.8 |  |
|  | $38,000-$47,999 | 859 | 22.7 | 501 | 22.7 | 358 | 22.5 |  | 482 | 23.0 | 241 | 23.0 | 241 | 23.0 |  |
|  | <$38,000 | 654 | 17.2 | 388 | 17.6 | 266 | 16.7 |  | 379 | 18.0 | 191 | 18.2 | 188 | 17.9 |  |
|  | Unknown | 69 | 1.8 | 40 | 1.8 | 29 | 1.8 |  |  |  |  |  |  |  |  |
| Educational: Non-High school | <7% | 970 | 25.6 | 548 | 24.9 | 422 | 26.6 | 0.064 | 528 | 25.1 | 266 | 25.3 | 262 | 25.0 | 0.641 |
|  | 7-12.9% | 1,225 | 32.3 | 722 | 32.8 | 503 | 31.7 |  | 694 | 33.0 | 338 | 32.2 | 356 | 33.9 |  |
|  | 13-20.9% | 917 | 24.2 | 513 | 23.3 | 404 | 25.4 |  | 520 | 24.8 | 257 | 24.5 | 263 | 25.0 |  |
|  | ≥21% | 618 | 16.3 | 384 | 17.4 | 234 | 14.7 |  | 358 | 17.0 | 189 | 18.0 | 169 | 16.1 |  |
|  | Unknown | 62 | 1.6 | 36 | 1.6 | 26 | 1.6 |  |  |  |  |  |  |  |  |
| Living location | Metro area | 1,864 | 49.2 | 1,110 | 50.4 | 754 | 47.5 | 0.249 | 1,015 | 48.3 | 502 | 47.8 | 513 | 48.9 | 0.9000 |
|  | Smaller metro area | 1,171 | 30.9 | 675 | 30.6 | 496 | 31.2 |  | 690 | 32.9 | 348 | 33.1 | 342 | 32.6 |  |
|  | Urban area | 552 | 14.6 | 306 | 13.9 | 246 | 15.5 |  | 323 | 15.4 | 167 | 15.9 | 156 | 14.9 |  |
|  | Rural area | 61 | 1.6 | 30 | 1.4 | 31 | 2.0 |  | 29 | 1.4 | 13 | 1.2 | 16 | 1.5 |  |
|  | Unknown | 144 | 3.8 | 82 | 3.7 | 62 | 3.9 |  | 43 | 2.0 | 20 | 1.9 | 23 | 2.2 |  |
| Distance to hospital | ≤10 | 1,093 | 28.8 | 678 | 30.8 | 415 | 26.1 | 0.011 | 588 | 28.0 | 292 | 27.8 | 296 | 28.2 | 0.994 |
|  | 11-20 | 678 | 17.9 | 402 | 18.2 | 276 | 17.4 |  | 367 | 17.5 | 183 | 17.4 | 184 | 17.5 |  |
|  | 21-50 | 768 | 20.3 | 420 | 19.1 | 348 | 21.9 |  | 443 | 21.1 | 221 | 21.0 | 222 | 21.1 |  |
|  | >50 | 1,191 | 31.4 | 667 | 30.3 | 524 | 33.0 |  | 702 | 33.4 | 354 | 33.7 | 348 | 33.1 |  |
|  | Unknown | 62 | 1.6 | 36 | 1.6 | 26 | 1.6 |  |  |  |  |  |  |  |  |
| Comorbidity | 0 | 3,068 | 80.9 | 1,770 | 80.3 | 1,298 | 81.7 | 0.452 | 1,711 | 81.5 | 853 | 81.2 | 858 | 81.7 | 0.748 |
|  | 1 | 593 | 15.6 | 351 | 15.9 | 242 | 15.2 |  | 325 | 15.5 | 162 | 15.4 | 163 | 15.5 |  |
|  | 2 | 131 | 3.5 | 82 | 3.7 | 49 | 3.1 |  | 64 | 3.0 | 35 | 3.3 | 29 | 2.8 |  |
| Transition in care | No | 571 | 15.1 | 332 | 15.1 | 239 | 15.0 | <.001 | 323 | 15.4 | 164 | 15.6 | 159 | 15.1 | 0.955 |
|  | Yes | 1,962 | 51.7 | 1,076 | 48.8 | 886 | 55.8 |  | 1,126 | 53.6 | 561 | 53.4 | 565 | 53.8 |  |
|  | Unknown | 1,259 | 33.2 | 795 | 36.1 | 464 | 29.2 |  | 651 | 31.0 | 325 | 31.0 | 326 | 31.0 |  |
| Date of diagnosis | 2004-2005 | 620 | 16.4 | 400 | 18.2 | 220 | 13.8 | <.001 | 318 | 15.1 | 162 | 15.4 | 156 | 14.9 | 0.991 |
|  | 2006-2007 | 671 | 17.7 | 414 | 18.8 | 257 | 16.2 |  | 377 | 18.0 | 190 | 18.1 | 187 | 17.8 |  |
|  | 2008-2009 | 825 | 21.8 | 499 | 22.7 | 326 | 20.5 |  | 444 | 21.1 | 221 | 21.0 | 223 | 21.2 |  |
|  | 2010-2011 | 803 | 21.2 | 441 | 20.0 | 362 | 22.8 |  | 463 | 22.0 | 232 | 22.1 | 231 | 22.0 |  |
|  | 2012-2013 | 873 | 23.0 | 449 | 20.4 | 424 | 26.7 |  | 498 | 23.7 | 245 | 23.3 | 253 | 24.1 |  |
| Primary site | Upper limb | 871 | 23.0 | 541 | 24.6 | 330 | 20.8 | 0.006 | 460 | 21.9 | 221 | 21.0 | 239 | 22.8 | 0.342 |
|  | Lower limb | 2,921 | 77.0 | 1,662 | 75.4 | 1,259 | 79.2 |  | 1,640 | 78.1 | 829 | 79.0 | 811 | 77.2 |  |
| Tumor size (cm) | ≤5 | 1,052 | 27.7 | 846 | 38.4 | 206 | 13.0 | <.001 | 381 | 18.1 | 188 | 17.9 | 193 | 18.4 | 0.962 |
|  | 5.01-10 | 1,373 | 36.2 | 743 | 33.7 | 630 | 39.6 |  | 877 | 41.8 | 437 | 41.6 | 440 | 41.9 |  |
|  | 10.01-15 | 726 | 19.1 | 325 | 14.8 | 401 | 25.2 |  | 457 | 21.8 | 228 | 21.7 | 229 | 21.8 |  |
|  | >15 | 641 | 16.9 | 289 | 13.1 | 352 | 22.2 |  | 385 | 18.3 | 197 | 18.8 | 188 | 17.9 |  |
| Clinical tumor stage | I | 791 | 20.9 | 633 | 28.7 | 158 | 9.9 | <.001 | 308 | 14.7 | 154 | 14.7 | 154 | 14.7 | 0.848 |
|  | II | 2,290 | 60.4 | 1,019 | 46.3 | 1,271 | 80.0 |  | 1,499 | 71.4 | 754 | 71.8 | 745 | 71.0 |  |
|  | Unknown | 711 | 18.8 | 551 | 25.0 | 160 | 10.1 |  | 293 | 14.0 | 142 | 13.5 | 151 | 14.4 |  |
| Depth of extension | Superficial | 935 | 24.7 | 731 | 33.2 | 204 | 12.8 | <.001 | 390 | 18.6 | 198 | 18.9 | 192 | 18.3 | 0.905 |
|  | Deep | 2,535 | 66.9 | 1,250 | 56.7 | 1,285 | 80.9 |  | 1,556 | 74.1 | 777 | 74.0 | 779 | 74.2 |  |
|  | Unknown | 322 | 8.5 | 222 | 10.1 | 100 | 6.3 |  | 154 | 7.3 | 75 | 7.1 | 79 | 7.5 |  |
| Chemotherapy | None | 2,770 | 73.0 | 1,762 | 80.0 | 1,008 | 63.4 | <.001 | 1,479 | 70.4 | 743 | 70.8 | 736 | 70.1 | 0.929 |
|  | Given | 916 | 24.2 | 373 | 16.9 | 543 | 34.2 |  | 563 | 26.8 | 279 | 26.6 | 284 | 27.0 |  |
|  | Unknown | 106 | 2.8 | 68 | 3.1 | 38 | 2.4 |  | 58 | 2.8 | 28 | 2.7 | 30 | 2.9 |  |
| Surgery margin | Negative | 3,228 | 85.1 | 1,815 | 82.4 | 1,413 | 88.9 | <.001 | 1,813 | 86.3 | 908 | 86.5 | 905 | 86.2 | 0.942 |
|  | Positive but unspecified extent | 147 | 3.9 | 106 | 4.8 | 41 | 2.6 |  | 80 | 3.8 | 42 | 4.0 | 38 | 3.6 |  |
|  | Microscopic residual | 253 | 6.7 | 186 | 8.4 | 67 | 4.2 |  | 116 | 5.5 | 58 | 5.5 | 58 | 5.5 |  |
|  | Macroscopic residual | 17 | 0.4 | 12 | 0.5 | 5 | 0.3 |  | 7 | 0.3 | 3 | 0.3 | 4 | 0.4 |  |
|  | Unknown | 147 | 3.9 | 84 | 3.8 | 63 | 4.0 |  | 84 | 4.0 | 39 | 3.7 | 45 | 4.3 |  |
| Facility volume | ≤7 (Lowest 25%) | 768 | 20.3 | 522 | 23.7 | 246 | 15.5 | <.001 | 367 | 17.5 | 184 | 17.5 | 183 | 17.4 | 0.838 |
|  | 8-81 (Middle 50% | 2,008 | 53.0 | 1,172 | 53.2 | 836 | 52.6 |  | 1,145 | 54.5 | 578 | 55.0 | 567 | 54.0 |  |
|  | >81 (Highest 25%) | 1,016 | 26.8 | 509 | 23.1 | 507 | 31.9 |  | 588 | 28.0 | 288 | 27.4 | 300 | 28.6 |  |
| Abbreviations: Limb-sparing surgery (LSS), radiotherapy (RT), pre-operative RT (pre-RT), post-operative RT (post-RT), centimeters (cm), American Joint Committee on Cancer (AJCC).  ^1^P-value from chi-square test | | | | | | | | | | | | | | | |

| **Supplemental Table 4.** Demographics and clinical characteristics of included high-grade extremity sarcoma patients by treatment (limb-sparing surgery alone versus pre-operative radiotherapy + limb-sparing surgery) before and after 1:1 matching the SEER Program | | | | | | | | | | | | | | | | |
| --- | --- | --- | --- | --- | --- | --- | --- | --- | --- | --- | --- | --- | --- | --- | --- | --- |
|  | Unmatched | | | | | | | Matched (1:1) | | | | | | | | |
| Variable | Total | | Treatment | | | |  | | Total | | | Treatment | | | |  |
|  |  |  | LSS | | Pre-RT + LSS | |  | |  |  |  | LSS | | Pre-RT + LSS | |  |
|  | N | % | N | % | N | % | P^1^ | | N | | % | N | % | N | % | P^1^ |
| Total | 1,259 | 100.0 | 775 | 100.0 | 484 | 100.0 |  | 674 | | 100.0 | | 337 | 100.0 | 337 | 100.0 |  |
| Facility location |  |  |  |  |  |  |  |  | |  | |  |  |  |  |  |
| Northeast | 198 | 15.7 | 145 | 18.7 | 53 | 11.0 | <.001 | 102 | | 15.1 | | 54 | 16.0 | 48 | 14.2 | 0.813 |
| South | 341 | 27.1 | 157 | 20.3 | 184 | 38.0 |  | 177 | | 26.3 | | 84 | 24.9 | 93 | 27.6 |  |
| Midwest | 110 | 8.7 | 72 | 9.3 | 38 | 7.9 |  | 60 | | 8.9 | | 29 | 8.6 | 31 | 9.2 |  |
| West | 610 | 48.5 | 401 | 51.7 | 209 | 43.2 |  | 335 | | 49.7 | | 170 | 50.4 | 165 | 49.0 |  |
| Age in years |  |  |  |  |  |  |  |  | |  | |  |  |  |  |  |
| ≥40 | 183 | 14.5 | 116 | 15.0 | 67 | 13.8 | <.001 | 101 | | 15.0 | | 51 | 15.1 | 50 | 14.8 | 0.975 |
| 41-50 | 194 | 15.4 | 118 | 15.2 | 76 | 15.7 |  | 122 | | 18.1 | | 63 | 18.7 | 59 | 17.5 |  |
| 51-60 | 255 | 20.3 | 143 | 18.5 | 112 | 23.1 |  | 125 | | 18.5 | | 64 | 19.0 | 61 | 18.1 |  |
| 61-70 | 258 | 20.5 | 141 | 18.2 | 117 | 24.2 |  | 144 | | 21.4 | | 69 | 20.5 | 75 | 22.3 |  |
| >70 | 369 | 29.3 | 257 | 33.2 | 112 | 23.1 |  | 182 | | 27.0 | | 90 | 26.7 | 92 | 27.3 |  |
| Sex |  |  |  |  |  |  |  |  | |  | |  |  |  |  |  |
| Male | 704 | 55.9 | 431 | 55.6 | 273 | 56.4 | 0.783 | 387 | | 57.4 | | 189 | 56.1 | 198 | 58.8 | 0.483 |
| Female | 555 | 44.1 | 344 | 44.4 | 211 | 43.6 |  | 287 | | 42.6 | | 148 | 43.9 | 139 | 41.2 |  |
| Marital status |  |  |  |  |  |  |  |  | |  | |  |  |  |  |  |
| Single (never married) | 225 | 17.9 | 142 | 18.3 | 83 | 17.1 | 0.026 | 127 | | 18.8 | | 61 | 18.1 | 66 | 19.6 | 0.892 |
| Married (including common law)/Domestic Partner | 719 | 57.1 | 418 | 53.9 | 301 | 62.2 |  | 390 | | 57.9 | | 199 | 59.1 | 191 | 56.7 |  |
| Separated/Divorced | 111 | 8.8 | 74 | 9.5 | 37 | 7.6 |  | 56 | | 8.3 | | 25 | 7.4 | 31 | 9.2 |  |
| Widowed | 162 | 12.9 | 109 | 14.1 | 53 | 11.0 |  | 81 | | 12.0 | | 42 | 12.5 | 39 | 11.6 |  |
| Unknown | 42 | 3.3 | 32 | 4.1 | 10 | 2.1 |  | 20 | | 3.0 | | 10 | 3.0 | 10 | 3.0 |  |
| Race/ethnicity |  |  |  |  |  |  |  |  | |  | |  |  |  |  |  |
| White Non-Hispanic | 876 | 69.6 | 529 | 68.3 | 347 | 71.7 | 0.018 | 472 | | 70.0 | | 234 | 69.4 | 238 | 70.6 | 0.770 |
| White Hispanic | 148 | 11.8 | 102 | 13.2 | 46 | 9.5 |  | 77 | | 11.4 | | 40 | 11.9 | 37 | 11.0 |  |
| Black | 126 | 10.0 | 68 | 8.8 | 58 | 12.0 |  | 74 | | 11.0 | | 40 | 11.9 | 34 | 10.1 |  |
| Other/Unknown | 109 | 8.7 | 76 | 9.8 | 33 | 6.8 |  | 51 | | 7.6 | | 23 | 6.8 | 28 | 8.3 |  |
| Insurance status |  |  |  |  |  |  |  |  | |  | |  |  |  |  |  |
| Not Insured | 49 | 3.9 | 33 | 4.3 | 16 | 3.3 | 0.004 | 30 | | 4.5 | | 21 | 6.2 | 9 | 2.7 | 0.042 |
| Insured/no specifics | 780 | 62.0 | 450 | 58.1 | 330 | 68.2 |  | 416 | | 61.7 | | 199 | 59.1 | 217 | 64.4 |  |
| Medicaid | 81 | 6.4 | 57 | 7.4 | 24 | 5.0 |  | 49 | | 7.3 | | 30 | 8.9 | 19 | 5.6 |  |
| Other/Unknown | 349 | 27.7 | 235 | 30.3 | 114 | 23.6 |  | 179 | | 26.6 | | 87 | 25.8 | 92 | 27.3 |  |
| Median household income |  |  |  |  |  |  |  |  | |  | |  |  |  |  |  |
| ≥$63,000 | 464 | 36.9 | 296 | 38.2 | 168 | 34.7 | 0.273 | 245 | | 36.4 | | 123 | 36.5 | 122 | 36.2 | 0.991 |
| $48,000-$62,999 | 525 | 41.7 | 324 | 41.8 | 201 | 41.5 |  | 282 | | 41.8 | | 141 | 41.8 | 141 | 41.8 |  |
| $38,000-$47,999 | 180 | 14.3 | 107 | 13.8 | 73 | 15.1 |  | 105 | | 15.6 | | 53 | 15.7 | 52 | 15.4 |  |
| <$38,000 | 90 | 7.1 | 48 | 6.2 | 42 | 8.7 |  | 42 | | 6.2 | | 20 | 5.9 | 22 | 6.5 |  |
| Educational: Non-High school |  |  |  |  |  |  |  |  | |  | |  |  |  |  |  |
| <7% | 66 | 5.2 | 40 | 5.2 | 26 | 5.4 | 0.840 | 35 | | 5.2 | | 16 | 4.7 | 19 | 5.6 | 0.898 |
| 7-12.9% | 458 | 36.4 | 275 | 35.5 | 183 | 37.8 |  | 230 | | 34.1 | | 114 | 33.8 | 116 | 34.4 |  |
| 13-20.9% | 424 | 33.7 | 264 | 34.1 | 160 | 33.1 |  | 242 | | 35.9 | | 125 | 37.1 | 117 | 34.7 |  |
| ≥21% | 311 | 24.7 | 196 | 25.3 | 115 | 23.8 |  | 167 | | 24.8 | | 82 | 24.3 | 85 | 25.2 |  |
| Living location |  |  |  |  |  |  |  |  | |  | |  |  |  |  |  |
| Metro | 783 | 62.2 | 491 | 63.4 | 292 | 60.3 | 0.604 | 420 | | 62.3 | | 213 | 63.2 | 207 | 61.4 | 0.944 |
| Small metro | 316 | 25.1 | 189 | 24.4 | 127 | 26.2 |  | 175 | | 26.0 | | 84 | 24.9 | 91 | 27.0 |  |
| Urban | 145 | 11.5 | 86 | 11.1 | 59 | 12.2 |  | 69 | | 10.2 | | 35 | 10.4 | 34 | 10.1 |  |
| Rural | 13 | 1.0 | 7 | 0.9 | 6 | 1.2 |  | 10 | | 1.5 | | 5 | 1.5 | 5 | 1.5 |  |
| Unknown | 2 | 0.2 | 2 | 0.3 | - | - |  |  | |  | |  |  |  |  |  |
| Date of diagnosis |  |  |  |  |  |  |  |  | |  | |  |  |  |  |  |
| 2004-2005 | 209 | 16.6 | 143 | 18.5 | 66 | 13.6 | 0.003 | 102 | | 15.1 | | 48 | 14.2 | 54 | 16.0 | 0.877 |
| 2006-2007 | 230 | 18.3 | 149 | 19.2 | 81 | 16.7 |  | 134 | | 19.9 | | 69 | 20.5 | 65 | 19.3 |  |
| 2008-2009 | 263 | 20.9 | 173 | 22.3 | 90 | 18.6 |  | 131 | | 19.4 | | 63 | 18.7 | 68 | 20.2 |  |
| 2010-2011 | 276 | 21.9 | 158 | 20.4 | 118 | 24.4 |  | 157 | | 23.3 | | 83 | 24.6 | 74 | 22.0 |  |
| 2012-2013 | 281 | 22.3 | 152 | 19.6 | 129 | 26.7 |  | 150 | | 22.3 | | 74 | 22.0 | 76 | 22.6 |  |
| Primary site |  |  |  |  |  |  |  |  | |  | |  |  |  |  |  |
| Upper limb | 307 | 24.4 | 204 | 26.3 | 103 | 21.3 | 0.043 | 151 | | 22.4 | | 68 | 20.2 | 83 | 24.6 | 0.166 |
| Lower limb | 952 | 75.6 | 571 | 73.7 | 381 | 78.7 |  | 523 | | 77.6 | | 269 | 79.8 | 254 | 75.4 |  |
| Tumor size (cm) |  |  |  |  |  |  |  |  | |  | |  |  |  |  |  |
| ≤5 | 461 | 36.6 | 385 | 49.7 | 76 | 15.7 | <.001 | 150 | | 22.3 | | 75 | 22.3 | 75 | 22.3 | 0.997 |
| 5.01-10 | 399 | 31.7 | 221 | 28.5 | 178 | 36.8 |  | 270 | | 40.1 | | 134 | 39.8 | 136 | 40.4 |  |
| 10.01-15 | 225 | 17.9 | 95 | 12.3 | 130 | 26.9 |  | 140 | | 20.8 | | 70 | 20.8 | 70 | 20.8 |  |
| >15 | 174 | 13.8 | 74 | 9.5 | 100 | 20.7 |  | 114 | | 16.9 | | 58 | 17.2 | 56 | 16.6 |  |
| AJCC tumor stage |  |  |  |  |  |  |  |  | |  | |  |  |  |  |  |
| II | 620 | 49.2 | 491 | 63.4 | 129 | 26.7 | <.001 | 235 | | 34.9 | | 114 | 33.8 | 121 | 35.9 | 0.572 |
| III | 639 | 50.8 | 284 | 36.6 | 355 | 73.3 |  | 439 | | 65.1 | | 223 | 66.2 | 216 | 64.1 |  |
| Depth of extension |  |  |  |  |  |  |  |  | |  | |  |  |  |  |  |
| Superficial | 341 | 27.1 | 269 | 34.7 | 72 | 14.9 | <.001 | 124 | | 18.4 | | 59 | 17.5 | 65 | 19.3 | 0.633 |
| Deep | 808 | 64.2 | 407 | 52.5 | 401 | 82.9 |  | 524 | | 77.7 | | 263 | 78.0 | 261 | 77.4 |  |
| Unknown | 110 | 8.7 | 99 | 12.8 | 11 | 2.3 |  | 26 | | 3.9 | | 15 | 4.5 | 11 | 3.3 |  |
| Abbreviations: Surveillance, Epidemiology, and End Results (SEER), limb-sparing surgery (LSS), radiation therapy (RT), pre-operative RT (pre-RT), post-operative RT (post-RT), centimeter (cm)  ^1^P-value from chi-squared test. | | | | | | | | | | | | | | | | |

| **Supplemental Table 5.** Multivariable regression on survival outcomes using National Cancer Database (NCDB) and Surveillance, Epidemiology, and End Results (SEER) Program | | | | |
| --- | --- | --- | --- | --- |
| **Data Set** | **Outcome** | **Treatment** | **Hazard Ratios (95%CI)** | **P-value^1^** |
| NCDB | Overall Survival | LSS | Reference |  |
|  |  | Pre-RT+ LSS | 0.67 (0.58, 0.78) | <.001 |
| NCDB | Overall Survival | LSS | Reference |  |
|  |  | LSS + post-RT | 0.71 (0.64, 0.78) | <.001 |
| SEER | Overall Survival | LSS | Reference |  |
|  |  | Pre-RT+ LSS | 0.57 (0.43, 0.75) | 0.003 |
| SEER | Overall Survival | LSS | Reference |  |
|  |  | LSS + post-RT | 0.71 (0.59, 0.86) | <.001 |
| SEER | Sarcoma Mortality | LSS | Reference |  |
|  |  | Pre-RT+ LSS | 0.69 (0.49, 0.95) | 0.025 |
| SEER | Sarcoma Mortality | LSS | Reference |  |
|  |  | LSS + post-RT | 0.86 (0.68, 1.08) | 0.188 |
| Abbreviations: Confidence interval (CI), limb-sparing surgery (LSS), radiotherapy (RT), pre-operative RT (pre-RT), post-operative RT (post-RT).  ^1^ Adjusted for all covariates listed in Table 1. | | | | |

| **Supplemental Table 6.** Demographics and clinical characteristics of included high-grade extremity sarcoma patients by treatment  (limb-sparing surgery alone versus limb-sparing surgery + post-operative radiotherapy) from the National Cancer Database (NCDB) | | | | | | | | | | | | | | | |
| --- | --- | --- | --- | --- | --- | --- | --- | --- | --- | --- | --- | --- | --- | --- | --- |
|  | Category | Unmatched | | | | | | | 1:1 matched | | | | | | |
|  |  | Total | | Treatment | | | |  | Total | | Treatment | | | |  |
| Variable |  |  |  | LSS | | LSS + post-RT | |  |  |  | LSS | | LSS + Post-RT | |  |
|  |  | N | % | N | % | N | % | P^1^ | N | % | N | % | N | % | P^1^ |
| Total |  | 5,762 | 100.0 | 2,203 | 100.0 | 3,559 | 100.0 |  | 3,966 | 100.0 | 1,983 | 100.0 | 1,983 | 100.0 |  |
| Facility type | Community Program | 1,776 | 30.8 | 582 | 26.4 | 1,194 | 33.5 | <.001 | 1,069 | 27.0 | 540 | 27.2 | 529 | 26.7 | 0.981 |
|  | Academic/Research Program | 2,655 | 46.1 | 1,060 | 48.1 | 1,595 | 44.8 |  | 1,934 | 48.8 | 965 | 48.7 | 969 | 48.9 |  |
|  | Integrated Network Cancer Program | 570 | 9.9 | 230 | 10.4 | 340 | 9.6 |  | 410 | 10.3 | 204 | 10.3 | 206 | 10.4 |  |
|  | Other/Unknown | 761 | 13.2 | 331 | 15.0 | 430 | 12.1 |  | 553 | 13.9 | 274 | 13.8 | 279 | 14.1 |  |
| Facility location | Northeast | 1,085 | 18.8 | 424 | 19.2 | 661 | 18.6 | <.001 | 781 | 19.7 | 387 | 19.5 | 394 | 19.9 | 0.997 |
|  | South | 1,636 | 28.4 | 685 | 31.1 | 951 | 26.7 |  | 1,196 | 30.2 | 601 | 30.3 | 595 | 30.0 |  |
|  | Midwest | 1,248 | 21.7 | 388 | 17.6 | 860 | 24.2 |  | 726 | 18.3 | 365 | 18.4 | 361 | 18.2 |  |
|  | West | 1,032 | 17.9 | 375 | 17.0 | 657 | 18.5 |  | 710 | 17.9 | 356 | 18.0 | 354 | 17.9 |  |
|  | Other/Unknown | 761 | 13.2 | 331 | 15.0 | 430 | 12.1 |  | 553 | 13.9 | 274 | 13.8 | 279 | 14.1 |  |
| Age in years | ≤40 | 812 | 14.1 | 346 | 15.7 | 466 | 13.1 | <.001 | 584 | 14.7 | 289 | 14.6 | 295 | 14.9 | 0.941 |
|  | 41-50 | 788 | 13.7 | 278 | 12.6 | 510 | 14.3 |  | 530 | 13.4 | 259 | 13.1 | 271 | 13.7 |  |
|  | 51-60 | 1,152 | 20.0 | 395 | 17.9 | 757 | 21.3 |  | 739 | 18.6 | 369 | 18.6 | 370 | 18.7 |  |
|  | 61-70 | 1,128 | 19.6 | 403 | 18.3 | 725 | 20.4 |  | 732 | 18.5 | 375 | 18.9 | 357 | 18.0 |  |
|  | >70 | 1,882 | 32.7 | 781 | 35.5 | 1,101 | 30.9 |  | 1,381 | 34.8 | 691 | 34.8 | 690 | 34.8 |  |
| Sex | Male | 3,045 | 52.8 | 1,133 | 51.4 | 1,912 | 53.7 | 0.090 | 2,073 | 52.3 | 1,030 | 51.9 | 1,043 | 52.6 | 0.679 |
|  | Female | 2,717 | 47.2 | 1,070 | 48.6 | 1,647 | 46.3 |  | 1,893 | 47.7 | 953 | 48.1 | 940 | 47.4 |  |
| Race/ethnicity | White Non-Hispanic | 4,239 | 73.6 | 1,604 | 72.8 | 2,635 | 74.0 | 0.360 | 2,903 | 73.2 | 1,449 | 73.1 | 1,454 | 73.3 | 0.957 |
|  | White Hispanic | 369 | 6.4 | 157 | 7.1 | 212 | 6.0 |  | 262 | 6.6 | 131 | 6.6 | 131 | 6.6 |  |
|  | Black | 553 | 9.6 | 213 | 9.7 | 340 | 9.6 |  | 391 | 9.9 | 193 | 9.7 | 198 | 10.0 |  |
|  | Other/Unknown | 601 | 10.4 | 229 | 10.4 | 372 | 10.5 |  | 410 | 10.3 | 210 | 10.6 | 200 | 10.1 |  |
| Insurance status | Private Insurance | 2,713 | 47.1 | 935 | 42.4 | 1,778 | 50.0 | <.001 | 1,738 | 43.8 | 865 | 43.6 | 873 | 44.0 | 0.939 |
|  | Not Insured | 230 | 4.0 | 109 | 4.9 | 121 | 3.4 |  | 175 | 4.4 | 90 | 4.5 | 85 | 4.3 |  |
|  | Medicaid | 320 | 5.6 | 130 | 5.9 | 190 | 5.3 |  | 242 | 6.1 | 117 | 5.9 | 125 | 6.3 |  |
|  | Medicare | 2,336 | 40.5 | 965 | 43.8 | 1,371 | 38.5 |  | 1,686 | 42.5 | 851 | 42.9 | 835 | 42.1 |  |
|  | Other/Unknown | 163 | 2.8 | 64 | 2.9 | 99 | 2.8 |  | 125 | 3.2 | 60 | 3.0 | 65 | 3.3 |  |
| Median income 2012 | ≥$63,000 | 1,918 | 33.3 | 699 | 31.7 | 1,219 | 34.3 | 0.019 | 1,337 | 33.7 | 664 | 33.5 | 673 | 33.9 | 0.981 |
|  | $48,000-$62,999 | 1,530 | 26.6 | 575 | 26.1 | 955 | 26.8 |  | 1,041 | 26.2 | 526 | 26.5 | 515 | 26.0 |  |
|  | $38,000-$47,999 | 1,307 | 22.7 | 501 | 22.7 | 806 | 22.6 |  | 921 | 23.2 | 460 | 23.2 | 461 | 23.2 |  |
|  | <$38,000 | 913 | 15.8 | 388 | 17.6 | 525 | 14.8 |  | 667 | 16.8 | 333 | 16.8 | 334 | 16.8 |  |
|  | Not Available | 94 | 1.6 | 40 | 1.8 | 54 | 1.5 |  |  |  |  |  |  |  |  |
| Educational: Non-High school | <7% | 1,503 | 26.1 | 548 | 24.9 | 955 | 26.8 | 0.157 | 1,025 | 25.8 | 511 | 25.8 | 514 | 25.9 | 0.956 |
|  | 7-12.9% | 1,890 | 32.8 | 722 | 32.8 | 1,168 | 32.8 |  | 1,310 | 33.0 | 663 | 33.4 | 647 | 32.6 |  |
|  | 13-20.9% | 1,350 | 23.4 | 513 | 23.3 | 837 | 23.5 |  | 950 | 24.0 | 470 | 23.7 | 480 | 24.2 |  |
|  | ≥21% | 935 | 16.2 | 384 | 17.4 | 551 | 15.5 |  | 681 | 17.2 | 339 | 17.1 | 342 | 17.2 |  |
|  | Unknown | 84 | 1.5 | 36 | 1.6 | 48 | 1.3 |  |  |  |  |  |  |  |  |
| Living location | Metro area | 2,962 | 51.4 | 1,110 | 50.4 | 1,852 | 52.0 | 0.595 | 2,106 | 53.1 | 1,047 | 52.8 | 1,059 | 53.4 | 0.968 |
|  | Smaller metro area | 1,712 | 29.7 | 675 | 30.6 | 1,037 | 29.1 |  | 1,158 | 29.2 | 589 | 29.7 | 569 | 28.7 |  |
|  | Urban area | 813 | 14.1 | 306 | 13.9 | 507 | 14.2 |  | 556 | 14.0 | 276 | 13.9 | 280 | 14.1 |  |
|  | Rural area | 70 | 1.2 | 30 | 1.4 | 40 | 1.1 |  | 56 | 1.4 | 27 | 1.4 | 29 | 1.5 |  |
|  | Unknown | 205 | 3.6 | 82 | 3.7 | 123 | 3.5 |  | 90 | 2.3 | 44 | 2.2 | 46 | 2.3 |  |
| Distance to hospital | ≤10 | 2,181 | 37.9 | 678 | 30.8 | 1,503 | 42.2 | <.001 | 1,318 | 33.2 | 663 | 33.4 | 655 | 33.0 | 0.966 |
|  | 11-20 | 1,090 | 18.9 | 402 | 18.2 | 688 | 19.3 |  | 784 | 19.8 | 383 | 19.3 | 401 | 20.2 |  |
|  | 21-50 | 1,113 | 19.3 | 420 | 19.1 | 693 | 19.5 |  | 805 | 20.3 | 402 | 20.3 | 403 | 20.3 |  |
|  | >50 | 1,295 | 22.5 | 667 | 30.3 | 628 | 17.6 |  | 1,057 | 26.7 | 534 | 26.9 | 523 | 26.4 |  |
|  | Unknown | 83 | 1.4 | 36 | 1.6 | 47 | 1.3 |  | 2 | 0.1 | 1 | 0.1 | 1 | 0.1 |  |
| Comorbidity | 0 | 4,757 | 82.6 | 1,770 | 80.3 | 2,987 | 83.9 | 0.002 | 3,232 | 81.5 | 1,614 | 81.4 | 1,618 | 81.6 | 0.851 |
|  | 1 | 825 | 14.3 | 351 | 15.9 | 474 | 13.3 |  | 597 | 15.1 | 303 | 15.3 | 294 | 14.8 |  |
|  | 2 | 180 | 3.1 | 82 | 3.7 | 98 | 2.8 |  | 137 | 3.5 | 66 | 3.3 | 71 | 3.6 |  |
| Transition in care | No | 739 | 12.8 | 332 | 15.1 | 407 | 11.4 | <.001 | 572 | 14.4 | 282 | 14.2 | 290 | 14.6 | 0.841 |
|  | Yes | 3,089 | 53.6 | 1,076 | 48.8 | 2,013 | 56.6 |  | 1,964 | 49.5 | 991 | 50.0 | 973 | 49.1 |  |
|  | Unknown | 1,934 | 33.6 | 795 | 36.1 | 1,139 | 32.0 |  | 1,430 | 36.1 | 710 | 35.8 | 720 | 36.3 |  |
| Date of diagnosis | 2004-2005 | 1,065 | 18.5 | 400 | 18.2 | 665 | 18.7 | 0.189 | 709 | 17.9 | 358 | 18.1 | 351 | 17.7 | 0.987 |
|  | 2006-2007 | 1,166 | 20.2 | 414 | 18.8 | 752 | 21.1 |  | 790 | 19.9 | 390 | 19.7 | 400 | 20.2 |  |
|  | 2008-2009 | 1,257 | 21.8 | 499 | 22.7 | 758 | 21.3 |  | 855 | 21.6 | 432 | 21.8 | 423 | 21.3 |  |
|  | 2010-2011 | 1,144 | 19.9 | 441 | 20.0 | 703 | 19.8 |  | 794 | 20.0 | 394 | 19.9 | 400 | 20.2 |  |
|  | 2012-2013 | 1,130 | 19.6 | 449 | 20.4 | 681 | 19.1 |  | 818 | 20.6 | 409 | 20.6 | 409 | 20.6 |  |
| Primary site | Upper limb | 1,463 | 25.4 | 541 | 24.6 | 922 | 25.9 | 0.253 | 1,012 | 25.5 | 508 | 25.6 | 504 | 25.4 | 0.884 |
|  | Lower limb | 4,299 | 74.6 | 1,662 | 75.4 | 2,637 | 74.1 |  | 2,954 | 74.5 | 1,475 | 74.4 | 1,479 | 74.6 |  |
| Tumor size (cm) | ≤5 | 1,935 | 33.6 | 846 | 38.4 | 1,089 | 30.6 | <.001 | 1,461 | 36.8 | 726 | 36.6 | 735 | 37.1 | 0.983 |
|  | 5.01-10 | 2,116 | 36.7 | 743 | 33.7 | 1,373 | 38.6 |  | 1,389 | 35.0 | 694 | 35.0 | 695 | 35.0 |  |
|  | 10.01-15 | 987 | 17.1 | 325 | 14.8 | 662 | 18.6 |  | 605 | 15.3 | 304 | 15.3 | 301 | 15.2 |  |
|  | >15 | 724 | 12.6 | 289 | 13.1 | 435 | 12.2 |  | 511 | 12.9 | 259 | 13.1 | 252 | 12.7 |  |
| Clinical tumor stage | I | 1,419 | 24.6 | 633 | 28.7 | 786 | 22.1 | <.001 | 1,092 | 27.5 | 542 | 27.3 | 550 | 27.7 | 0.960 |
|  | II | 2,790 | 48.4 | 1,019 | 46.3 | 1,771 | 49.8 |  | 1,861 | 46.9 | 933 | 47.0 | 928 | 46.8 |  |
|  | Unknown | 1,553 | 27.0 | 551 | 25.0 | 1,002 | 28.2 |  | 1,013 | 25.5 | 508 | 25.6 | 505 | 25.5 |  |
| Depth of extension | Superficial | 1,702 | 29.5 | 731 | 33.2 | 971 | 27.3 | <.001 | 1,276 | 32.2 | 636 | 32.1 | 640 | 32.3 | 0.987 |
|  | Deep | 3,551 | 61.6 | 1,250 | 56.7 | 2,301 | 64.7 |  | 2,313 | 58.3 | 1,159 | 58.4 | 1,154 | 58.2 |  |
|  | Unknown | 509 | 8.8 | 222 | 10.1 | 287 | 8.1 |  | 377 | 9.5 | 188 | 9.5 | 189 | 9.5 |  |
| Chemotherapy | None | 4,433 | 76.9 | 1,762 | 80.0 | 2,671 | 75.0 | <.001 | 3,147 | 79.3 | 1,571 | 79.2 | 1,576 | 79.5 | 0.932 |
|  | Given | 1,163 | 20.2 | 373 | 16.9 | 790 | 22.2 |  | 697 | 17.6 | 349 | 17.6 | 348 | 17.5 |  |
|  | Unknown | 166 | 2.9 | 68 | 3.1 | 98 | 2.8 |  | 122 | 3.1 | 63 | 3.2 | 59 | 3.0 |  |
| Surgery margin | Negative | 4,592 | 79.7 | 1,815 | 82.4 | 2,777 | 78.0 | <.001 | 3,232 | 81.5 | 1,612 | 81.3 | 1,620 | 81.7 | 0.964 |
|  | Positive but unspecified extent | 325 | 5.6 | 106 | 4.8 | 219 | 6.2 |  | 202 | 5.1 | 101 | 5.1 | 101 | 5.1 |  |
|  | Microscopic residual | 580 | 10.1 | 186 | 8.4 | 394 | 11.1 |  | 365 | 9.2 | 182 | 9.2 | 183 | 9.2 |  |
|  | Macroscopic residual | 42 | 0.7 | 12 | 0.5 | 30 | 0.8 |  | 24 | 0.6 | 12 | 0.6 | 12 | 0.6 |  |
|  | Unknown | 223 | 3.9 | 84 | 3.8 | 139 | 3.9 |  | 143 | 3.6 | 76 | 3.8 | 67 | 3.4 |  |
| Facility volume | ≤7 (Lowest 25%) | 1,667 | 28.9 | 522 | 23.7 | 1,145 | 32.2 | <.001 | 983 | 24.8 | 489 | 24.7 | 494 | 24.9 | 0.951 |
|  | 8-81 (Middle 50% | 2,835 | 49.2 | 1,172 | 53.2 | 1,663 | 46.7 |  | 2,058 | 51.9 | 1,034 | 52.1 | 1,024 | 51.6 |  |
|  | >81 (Highest 25%) | 1,260 | 21.9 | 509 | 23.1 | 751 | 21.1 |  | 925 | 23.3 | 460 | 23.2 | 465 | 23.4 |  |
| Abbreviations: Limb-sparing surgery (LSS), radiation therapy (RT), post-operative RT, centimeter (cm)  ^1^ Chi-square test. | | | | | | | | | | | | | | | |

| **Supplemental Table 7.** Demographics and clinical characteristics of included high-grade extremity sarcoma patients by treatment from the Surveillance, Epidemiology, and End Results (SEER) Program | | | | | | | | | | | | | | |
| --- | --- | --- | --- | --- | --- | --- | --- | --- | --- | --- | --- | --- | --- | --- |
|  | Unmatched | | | | | | | Matched (1:1) | | | | | | |
| Variable | Total | | Treatment | | | |  | Total | | Treatment | | | |  |
|  |  |  | LSS | | LSS + post-RT | |  |  |  | LSS | | LSS + post-RT | |  |
|  | N | % | N | % | N | % | P^1^ | N | % | N | % | N | % | P^1^ |
| Total | 2,285 | 100.0 | 775 | 100.0 | 1,510 | 100.0 |  | 1,498 | 100.0 | 749 | 100.0 | 749 | 100.0 |  |
| Facility location |  |  |  |  |  |  |  |  |  |  |  |  |  |  |
| Northeast | 375 | 16.4 | 145 | 18.7 | 230 | 15.2 | 0.001 | 273 | 18.2 | 140 | 18.7 | 133 | 17.8 | 0.893 |
| South | 391 | 17.1 | 157 | 20.3 | 234 | 15.5 |  | 295 | 19.7 | 148 | 19.8 | 147 | 19.6 |  |
| Midwest | 219 | 9.6 | 72 | 9.3 | 147 | 9.7 |  | 148 | 9.9 | 70 | 9.3 | 78 | 10.4 |  |
| West | 1,300 | 56.9 | 401 | 51.7 | 899 | 59.5 |  | 782 | 52.2 | 391 | 52.2 | 391 | 52.2 |  |
| Age in years |  |  |  |  |  |  |  |  |  |  |  |  |  |  |
| ≤40 | 329 | 14.4 | 116 | 15.0 | 213 | 14.1 | 0.545 | 226 | 15.1 | 113 | 15.1 | 113 | 15.1 | 0.652 |
| 41-50 | 354 | 15.5 | 118 | 15.2 | 236 | 15.6 |  | 247 | 16.5 | 114 | 15.2 | 133 | 17.8 |  |
| 51-60 | 450 | 19.7 | 143 | 18.5 | 307 | 20.3 |  | 284 | 19.0 | 139 | 18.6 | 145 | 19.4 |  |
| 61-70 | 437 | 19.1 | 141 | 18.2 | 296 | 19.6 |  | 269 | 18.0 | 138 | 18.4 | 131 | 17.5 |  |
| >70 | 715 | 31.3 | 257 | 33.2 | 458 | 30.3 |  | 472 | 31.5 | 245 | 32.7 | 227 | 30.3 |  |
| Sex |  |  |  |  |  |  |  |  |  |  |  |  |  |  |
| Male | 1,252 | 54.8 | 431 | 55.6 | 821 | 54.4 | 0.572 | 837 | 55.9 | 417 | 55.7 | 420 | 56.1 | 0.876 |
| Female | 1,033 | 45.2 | 344 | 44.4 | 689 | 45.6 |  | 661 | 44.1 | 332 | 44.3 | 329 | 43.9 |  |
| Marital status |  |  |  |  |  |  |  |  |  |  |  |  |  |  |
| Single (never married) | 413 | 18.1 | 142 | 18.3 | 271 | 17.9 | 0.006 | 274 | 18.3 | 141 | 18.8 | 133 | 17.8 | 0.925 |
| Married (including common law)/Domestic Partner | 1,326 | 58.0 | 418 | 53.9 | 908 | 60.1 |  | 828 | 55.3 | 410 | 54.7 | 418 | 55.8 |  |
| Separated/Divorced | 213 | 9.3 | 74 | 9.5 | 139 | 9.2 |  | 149 | 9.9 | 71 | 9.5 | 78 | 10.4 |  |
| Widowed | 262 | 11.5 | 109 | 14.1 | 153 | 10.1 |  | 191 | 12.8 | 99 | 13.2 | 92 | 12.3 |  |
| Unknown | 71 | 3.1 | 32 | 4.1 | 39 | 2.6 |  | 56 | 3.7 | 28 | 3.7 | 28 | 3.7 |  |
| Race/ethnicity |  |  |  |  |  |  |  |  |  |  |  |  |  |  |
| White Non-Hispanic | 1,577 | 69.0 | 529 | 68.3 | 1,048 | 69.4 | 0.936 | 1,013 | 67.6 | 512 | 68.4 | 501 | 66.9 | 0.804 |
| White Hispanic | 290 | 12.7 | 102 | 13.2 | 188 | 12.5 |  | 208 | 13.9 | 98 | 13.1 | 110 | 14.7 |  |
| Black | 201 | 8.8 | 68 | 8.8 | 133 | 8.8 |  | 133 | 8.9 | 65 | 8.7 | 68 | 9.1 |  |
| Other/Unknown | 217 | 9.5 | 76 | 9.8 | 141 | 9.3 |  | 144 | 9.6 | 74 | 9.9 | 70 | 9.3 |  |
| Insurance status |  |  |  |  |  |  |  |  |  |  |  |  |  |  |
| Not Insured | 64 | 2.8 | 33 | 4.3 | 31 | 2.1 | 0.002 | 47 | 3.1 | 33 | 4.4 | 14 | 1.9 | 0.009 |
| Insured/no specifics | 1,355 | 59.3 | 450 | 58.1 | 905 | 59.9 |  | 898 | 59.9 | 433 | 57.8 | 465 | 62.1 |  |
| Medicaid | 135 | 5.9 | 57 | 7.4 | 78 | 5.2 |  | 94 | 6.3 | 55 | 7.3 | 39 | 5.2 |  |
| Other/Unknown | 731 | 32.0 | 235 | 30.3 | 496 | 32.8 |  | 459 | 30.6 | 228 | 30.4 | 231 | 30.8 |  |
| Median household income |  |  |  |  |  |  |  |  |  |  |  |  |  |  |
| ≥$63,000 | 981 | 42.9 | 296 | 38.2 | 685 | 45.4 | <.001 | 582 | 38.9 | 293 | 39.1 | 289 | 38.6 | 0.995 |
| $48,000-$62,999 | 892 | 39.0 | 324 | 41.8 | 568 | 37.6 |  | 628 | 41.9 | 312 | 41.7 | 316 | 42.2 |  |
| $38,000-$47,999 | 320 | 14.0 | 107 | 13.8 | 213 | 14.1 |  | 209 | 14.0 | 104 | 13.9 | 105 | 14.0 |  |
| <$38,000 | 92 | 4.0 | 48 | 6.2 | 44 | 2.9 |  | 79 | 5.3 | 40 | 5.3 | 39 | 5.2 |  |
| Educational: Non-High school |  |  |  |  |  |  |  |  |  |  |  |  |  |  |
| <7% | 107 | 4.7 | 40 | 5.2 | 67 | 4.4 | 0.160 | 77 | 5.1 | 39 | 5.2 | 38 | 5.1 | 0.964 |
| 7-12.9% | 866 | 37.9 | 275 | 35.5 | 591 | 39.1 |  | 541 | 36.1 | 275 | 36.7 | 266 | 35.5 |  |
| 13-20.9% | 787 | 34.4 | 264 | 34.1 | 523 | 34.6 |  | 506 | 33.8 | 250 | 33.4 | 256 | 34.2 |  |
| ≥21% | 525 | 23.0 | 196 | 25.3 | 329 | 21.8 |  | 374 | 25.0 | 185 | 24.7 | 189 | 25.2 |  |
| Living location |  |  |  |  |  |  |  |  |  |  |  |  |  |  |
| Metro area | 1,516 | 66.3 | 491 | 63.4 | 1,025 | 67.9 | 0.008 | 969 | 64.7 | 478 | 63.8 | 491 | 65.6 | 0.956 |
| Small metro area | 558 | 24.4 | 189 | 24.4 | 369 | 24.4 |  | 362 | 24.2 | 185 | 24.7 | 177 | 23.6 |  |
| Urban area | 191 | 8.4 | 86 | 11.1 | 105 | 7.0 |  | 152 | 10.1 | 79 | 10.5 | 73 | 9.7 |  |
| Rural area | 17 | 0.7 | 7 | 0.9 | 10 | 0.7 |  | 13 | 0.9 | 6 | 0.8 | 7 | 0.9 |  |
| Unknown | 3 | 0.1 | 2 | 0.3 | 1 | 0.1 |  | 2 | 0.1 | 1 | 0.1 | 1 | 0.1 |  |
| Date of diagnosis |  |  |  |  |  |  |  |  |  |  |  |  |  |  |
| 2004-2005 | 463 | 20.3 | 143 | 18.5 | 320 | 21.2 | 0.423 | 290 | 19.4 | 139 | 18.6 | 151 | 20.2 | 0.909 |
| 2006-2007 | 452 | 19.8 | 149 | 19.2 | 303 | 20.1 |  | 277 | 18.5 | 144 | 19.2 | 133 | 17.8 |  |
| 2008-2009 | 471 | 20.6 | 173 | 22.3 | 298 | 19.7 |  | 331 | 22.1 | 165 | 22.0 | 166 | 22.2 |  |
| 2010-2011 | 459 | 20.1 | 158 | 20.4 | 301 | 19.9 |  | 304 | 20.3 | 151 | 20.2 | 153 | 20.4 |  |
| 2012-2013 | 440 | 19.3 | 152 | 19.6 | 288 | 19.1 |  | 296 | 19.8 | 150 | 20.0 | 146 | 19.5 |  |
| Primary site |  |  |  |  |  |  |  |  |  |  |  |  |  |  |
| Upper limb | 578 | 25.3 | 204 | 26.3 | 374 | 24.8 | 0.418 | 392 | 26.2 | 197 | 26.3 | 195 | 26.0 | 0.906 |
| Lower limb | 1,707 | 74.7 | 571 | 73.7 | 1,136 | 75.2 |  | 1,106 | 73.8 | 552 | 73.7 | 554 | 74.0 |  |
| Tumor size (cm) |  |  |  |  |  |  |  |  |  |  |  |  |  |  |
| ≤5 | 897 | 39.3 | 385 | 49.7 | 512 | 33.9 | <.001 | 726 | 48.5 | 360 | 48.1 | 366 | 48.9 | 0.639 |
| 5.01-10 | 756 | 33.1 | 221 | 28.5 | 535 | 35.4 |  | 448 | 29.9 | 220 | 29.4 | 228 | 30.4 |  |
| 10.01-15 | 365 | 16.0 | 95 | 12.3 | 270 | 17.9 |  | 174 | 11.6 | 95 | 12.7 | 79 | 10.5 |  |
| >15 | 267 | 11.7 | 74 | 9.5 | 193 | 12.8 |  | 150 | 10.0 | 74 | 9.9 | 76 | 10.1 |  |
| AJCC tumor stage |  |  |  |  |  |  |  |  |  |  |  |  |  |  |
| II | 1,231 | 53.9 | 491 | 63.4 | 740 | 49.0 | <.001 | 934 | 62.3 | 466 | 62.2 | 468 | 62.5 | 0.915 |
| III | 1,054 | 46.1 | 284 | 36.6 | 770 | 51.0 |  | 564 | 37.7 | 283 | 37.8 | 281 | 37.5 |  |
| Depth of extension |  |  |  |  |  |  |  |  |  |  |  |  |  |  |
| Superficial | 710 | 31.1 | 269 | 34.7 | 441 | 29.2 | <.001 | 527 | 35.2 | 264 | 35.2 | 263 | 35.1 | 0.996 |
| Deep | 1,378 | 60.3 | 407 | 52.5 | 971 | 64.3 |  | 808 | 53.9 | 404 | 53.9 | 404 | 53.9 |  |
| Unknown | 197 | 8.6 | 99 | 12.8 | 98 | 6.5 |  | 163 | 10.9 | 81 | 10.8 | 82 | 10.9 |  |
| Abbreviations: Limb-sparing surgery (LSS), radiation therapy (RT), post-operative RT (post-RT), centimeter (cm)  ^1^ Chi-square test. | | | | | | | | | | | | | | |
